# Evidence for the free radical/oxidative stress theory of ageing from the CHANCES consortium: a meta-analysis of individual participant data

**DOI:** 10.1186/s12916-015-0537-7

**Published:** 2015-12-15

**Authors:** Ben Schöttker, Hermann Brenner, Eugène HJM Jansen, Julian Gardiner, Anne Peasey, Růžena Kubínová, Andrzej Pająk, Roman Topor-Madry, Abdonas Tamosiunas, Kai-Uwe Saum, Bernd Holleczek, Hynek Pikhart, Martin Bobak

**Affiliations:** Division of Clinical Epidemiology and Ageing Research, German Cancer Research Center, Im Neuenheimer Feld 581, 69120 Heidelberg, Germany; Network Aging Research, University of Heidelberg, Bergheimer Strasse 20, 69120 Heidelberg, Germany; Laboratory for Health Protection Research, National Institute for Public Health and the Environment, PO Box 1, 3720 BA Bilthoven, The Netherlands; Department Epidemiology and Public Health, University College London, 1-19 Torrington Place, London, WC1E 6BT Great Britain; National Institute of Public Health, Prague, Czech Republic; Jagiellonian University Medical College, Faculty of Health Sciences, Krakow, Poland; Institute of Cardiology of Lithuanian University of Health Sciences, Kaunas, Lithuania; Saarland Cancer Registry, Präsident Baltz-Strasse 5, 66119 Saarbrücken, Germany

**Keywords:** Cancer mortality, Cardiovascular mortality, Cohort study, Death, Epidemiology, Free radicals, Meta-analysis, Mortality, Oxidative stress

## Abstract

**Background:**

The free radical/oxidative stress theory of ageing has received considerable attention, but the evidence on the association of oxidative stress markers with mortality is sparse.

**Methods:**

We measured derivatives of reactive oxygen metabolite (D-ROM) levels as a proxy for the reactive oxygen species concentration and total thiol levels (TTL) as a proxy for the redox control status in 10,622 men and women (age range, 45–85 years), from population-based cohorts from Germany, Poland, Czech Republic, and Lithuania, of whom 1,702 died during follow-up.

**Results:**

Both oxidative stress markers were significantly associated with all-cause mortality independently from established risk factors (including inflammation) and from each other in all cohorts. Regarding cause-specific mortality, compared to low D-ROM levels (≤340 Carr U), very high D-ROM levels (>500 Carr U) were strongly associated with both cardiovascular (relative risk (RR), 5.09; 95 % CI, 2.67–9.69) and cancer mortality (RR, 4.34; 95 % CI, 2.31–8.16). TTL was only associated with CVD mortality (RR, 1.30; 95 % CI, 1.15–1.48, for one-standard-deviation-decrease). The strength of the association of TTL with CVD mortality increased with age of the participants (RR for one-standard-deviation-decrease in those aged 70–85 years was 1.65; 95 % CI, 1.22–2.24).

**Conclusions:**

In these four population-based cohort studies from Central and Eastern Europe, the oxidative stress serum markers D-ROM and TTL were independently and strongly associated with all-cause and CVD mortality. In addition, D-ROM levels were also strongly associated with cancer mortality. This study provides epidemiological evidence supporting the free radical/oxidative stress theory of ageing and suggests that d-ROMs and TTL are useful oxidative stress markers associated with premature mortality.

**Electronic supplementary material:**

The online version of this article (doi:10.1186/s12916-015-0537-7) contains supplementary material, which is available to authorized users.

## Background

Ageing is a multi-factorial process. Most hypotheses on the underlying mechanisms of the ageing process involve deterioration of the maintenance of homeostatic metabolic, inflammatory, and/or redox processes in cells and tissues [1–3]. The free radical/oxidative stress theory of ageing puts an increased production of reactive oxygen species (ROS) in the centre of processes that promote cell ageing [4, 5]. ROS are natural products of mitochondrial energy synthesis. Oxidative stress occurs when the natural production of ROS cannot be balanced by the anti-oxidative capacity of tissues, leading primarily to mitochondrial DNA damage and dysfunction and higher cell apoptosis rates [4]. A higher apoptosis rate is associated with a higher cell turnover and a shortening of telomeres, the ends of the DNA that limit the maximal number of possible mitoses of mitotic cells [6]. In summary, an accelerated senescence of cells mediated by increased telomere loss following unbalanced ROS production could be one of the pathways in the multi-factorial process of ageing [7, 8].

The evidence from studies in humans for these hypotheses remains sparse. The main reason is that ROS have a very short half-life and cannot be directly measured in plasma or serum samples [9]. Recently, assays have been developed to measure indirect biomarkers of ROS that are stable enough to be assessed in stored blood samples. We have recently reported a pilot study with a small number of cases (n = 120 deaths) in which derivatives of reactive oxygen metabolites (d-ROMs) levels and total thiol levels (TTL) of proteins were independent predictors of all-cause mortality at older ages [10]. d-ROMs levels are a proxy for ROS production [11] and TTL are a proxy for the redox control status of blood. The d-ROMs assay detects hydroperoxide metabolites (chemical: R-O-O-H) mainly of lipids, but also of glycosides, amino acids, and proteins in the serum sample. Hydroperoxides are a stable class of d-ROMs that occur after reactions of the hydroxyl radical (chemical: OH*), a ROS produced mainly as a product of the respiratory chain reaction in mitochondria. Hydroperoxides are being transported from cells to blood vessels to decrease the oxidative potential of a cell and are therefore detectable in serum.

The TTL assay detects free thiol groups of the amino acids cysteine or methionine (chemical: R-S-H), which can be reversibly oxidized to disulfide bridges (chemical: R-S-S-R). The most frequent molecules in human blood with free thiol groups are glutathione, α-lipoic acid, and members of the thioredoxin protein family. These molecules are able to oppose the propagation phase of the peroxidation processes by building a plasma antioxidant barrier against free radicals in order to maintain redox homeostasis in human plasma [12].

This study investigated the association of d-ROMs levels and TTL with mortality from all-causes, cardiovascular disease (CVD), and cancer in an individual participant data meta-analysis of four large population-based cohort studies from Central and Eastern Europe. In addition, we also examined cross-sectional associations of d-ROMs levels and TTL with established risk factors for mortality.

## Methods

### Study population

This investigation is based on the HAPIEE cohorts (Health, Alcohol and Psychosocial factors in Eastern Europe) from Poland, Czech Republic, and Lithuania [13], and the 8-year follow-up of the German ESTHER cohort (German name: “Epidemiologische Studie zu Chancen der Verhütung, Früherkennung und optimierten Therapie chronischer Erkrankungen in der älteren Bevölkerung”) [10].

The total HAPIEE study comprises cohorts in four countries. As it was not possible to export blood samples from Russia, only three cohorts were included in the current analysis. Study participants were recruited in six Czech towns (Havirov/Karvina, Hradec Kralove, Jihlava, Kromeriz, Liberec, and Usti nad Labem) in 2002–2005 (n = 8,857), in Krakow, Poland, in 2002–2005 (n = 10,728), and in Kaunas, Lithuania, in 2006–2008 (n = 7,161). Each cohort consists of a random sample of men and women, aged 45–75 years at baseline, stratified by sex and 5-year age groups, selected from population registers. The overall response rate was 61 % [13]. Data were collected by questionnaire and a short examination in a clinic during which a fasting venous blood sample was taken.

The ESTHER study is a population-based cohort of 9,949 adults, aged 50–74 years at baseline, recruited by their general practitioners (GP) during a routine health check-up between 2000 and 2002 in the German federal state Saarland [14]. The current investigation is based on the 8-year follow-up, conducted between July 2008 and December 2010, at which study participants were between 56 and 85 years old. From baseline to 8-year follow-up, 499 individuals were deceased, 505 individuals were no longer able to participate due to poor health, and 680 had declined further participation. From the remaining 8,265 participants, 6,061 sent back a questionnaire (response rate, 73.4 %) and 4,637 donated a blood sample in the office of their GP. The GPs of the study participants also completed a questionnaire about the health status of the study participants (available for n = 5,997). Furthermore, 3,124 study participants consented to an additional 3-hour geriatric assessment performed by study physicians at the participant’s homes.

Variables in ESTHER and HAPIEE studies were harmonized and oxidative stress markers were measured in the framework of the Consortium on Health and Ageing: Network of Cohorts in Europe and the United States (CHANCES; www.chancesfp7.eu), which has been described elsewhere [15, 16]. The studies are conducted in accordance with the declaration of Helsinki and written informed consent was obtained from all study participants.

### Ethics, consent and permissions

The included studies were approved by the respective local ethics committees (ESTHER: Medical Faculty of the University of Heidelberg and the Medical Association of Saarland; HAPIEE: University College London (Great Britain), National Institute of Public Health (Prague, Czech Republic), Jagiellonian University (Krakow, Poland), and Lithuanian University of Health Sciences (Kaunas, Lithuania)). Written informed consent has been obtained from all participants included in the analysed studies and the studies are being conducted in accordance with the Declaration of Helsinki.

### Covariate assessment

In the ESTHER and HAPIEE studies, sociodemographic, lifestyle, and history of disease data were assessed by self-administered questionnaires. Height and weight were measured and supplemented by self-reported information in ESTHER for those study participants who did not consent to the home visit. In addition to self-reported information, the history of common chronic diseases was validated in the ESTHER study by consulting medical records or the Saarland cancer registry. Renal impairment was defined by an estimated glomerular filtration rate <60 mL/min/1.73 m^2^, calculated with the creatinine-based Chronic Kidney Disease Epidemiology Collaboration equation [17]. In the ESTHER cohort, serum creatinine concentrations were assessed with a kinetic Jaffé method on a Cobas 8000 C701 (analyte: CREJ2 3000, Roche). Total cholesterol and high-density lipoprotein cholesterol (HDL) were assessed in serum samples by enzymatic chromatography (analytes: Chol2 2100, HDLC3 450, Roche) and C-reactive protein (CRP) by immunoturbidimetry on the Cobas 8000 C701 (analyte: CRPL3 500, Roche). In HAPIEE, total and HDL cholesterol were also assessed with the same assay methodology and Roche analytes, but different autoanalyzers were used (HAPIEE Czech Republic and Lithuania: Roche Cobas Mira; HAPIEE Poland: Hitachi 917/Modular P). In addition, in all HAPIEE cohorts, serum creatinine and CRP were assessed with the same assay methodology as in ESTHER, but with analytes from a different supplier (Beckman-Coulter) and on a different autoanalyzer (LX-20 Pro, Beckman-Coulter).

### Mortality ascertainment

For these analyses, we used follow-up for total mortality by official country-wide (Czech Republic, Germany), local (Lithuania), or regional mortality registers (Poland) until December 31, 2010, in Poland, December 31, 2011, in Czech Republic and Lithuania, and until July 31, 2014, in Germany. Cause-specific mortality in Germany was available only for deaths that occurred until April 2, 2013. The registries were complete for all participants that did not move outside the covered region of the registry. Loss to follow-up due to migration was 4 % in the HAPIEE cohorts (combined) and 2 % in the ESTHER study. All deaths coded with ICD-10 codes I00-I99 were considered cardiovascular deaths and cancer deaths were defined by the ICD-10 codes C00-C97.

### Oxidative stress serum marker measurement

The assays used to measure d-ROMs levels (Diacron, Grosseto, Italy) and TTL (Rel Assay Diagnostics, Gaziantep, Turkey) were adapted to an autoanalyzer (LX20-Pro, Beckman-Coulter, Woerden, The Netherlands) at the Laboratory for Health Protection Research (Bilthoven, The Netherlands), as described previously [18]. The d-ROMs assay measures the hydroperoxide concentration in Carratelli Units (Carr U), named after the inventor of the assay, Mauro Carratelli. Each Carr U corresponds to 0.08 mg hydrogen peroxide (H_2_O_2_)/100 mL in the sample [19]. The TTL assay measures the concentration of free thiol groups in the sample in μmol/L.

Measurements were performed in serum samples that had been stored for approximately 3–10 years in freezers with –80 °C. d-ROMs and TTL have a good long-term stability under these conditions [18, 20]. In the ESTHER 8-year follow-up, d-ROMs and TTL were measured in all participants that donated blood samples at their GP’s offices (n = 4,637). However, measurements were conducted at two different time-points because funding was not available to measure all samples in the pilot study conducted in June 2012 [10]. To correct for potential shifts in the assays, 80 samples of time-point 1 (June 2012) were measured again at time-point 2 (September 2014) and linear regression equations were obtained and used to standardize the results from time-point 2 to the levels of time-point 1, when the measurements in the HAPIEE cohorts also took place (throughout the year 2012). Agreement of d-ROMs measurements was high (Spearman correlation coefficient r = 0.92 and difference of means = 15 Carr U) and therefore no corrections were applied. For TTL, an obvious shift in the assay results (r = 0.88 and difference of means = 81 μmol/L) was corrected by applying the following equation to all measurements of time-point 2: TTL_time-point2_ = 0.746 × TTL_time-point1_. Furthermore, we excluded 610 measurements in blood samples that did not fulfil strict haemolytic, icteric and lipemic quality criteria in the ESTHER study, leaving 4,027 for analysis in the present study. In the HAPIEE cohorts, the number of samples that did not fulfil these quality criteria was negligible.

### Analytical study sample

Because of limited funding, d-ROMs levels and TTL were not measured in all participants in the HAPIEE cohorts; instead, a matched case control design was adopted (Additional file 1: Figure S1). Cases were defined as all subjects that died during follow-up (n = 1,433) or experienced incident non-fatal myocardial infarction (MI) or stroke (n = 658). Controls (n = 4,396) were frequency matched to cases by sex and 5-year age groups. To correct the oversampling for MI and stroke in this mortality analysis, subjects with non-fatal MI or non-fatal stroke during follow-up were excluded from the cases in a first step. In order to add a representative sample of subjects with non-fatal incident MI or stroke events to the controls, in a second step, the observed proportion of non-fatal incident MI or stroke events in each age- and sex-specific stratum in the total cohort was calculated. In a third step, these proportions were multiplied by the sample sizes in each stratum in the controls with measured oxidative stress markers. In a fourth step, the resulting numbers were randomly selected in each stratum from the group of subjects with non-fatal incident MI or stroke and added to the controls, resulting in a pool of 4,552 eligible controls for the 1,433 deaths. In the final step, the pool of eligible controls was used to individually match exactly two controls to each death by cohort, sex, and age (±5 years).

### Statistical analyses

Differences in baseline characteristics between cases (death during follow-up) and controls were assessed with the χ^2^ test for categorical variables and the Wilcoxon rank-sum test for continuous variables. Cross-sectional associations of established risk factors for mortality with high oxidative stress levels were assessed with logistic regression models comprising all baseline characteristics modelled as categorical variables (Table 1). The dependent variable “high oxidative stress” was defined by two different definitions based on d-ROMs levels or TTL. For d-ROMs, according to the manufacturer’s instructions, a clinical cut-off of >400 Carr U was employed, which classified 23 % of the ESTHER cohort and 30 % of eligible controls of the HAPIEE cohort in the “high” oxidative stress category. For TTL, no clinical cut-offs are available and, as the assay is not yet standardized, population means varied strongly between cohorts. Therefore, to match the proportion of individuals with high oxidative stress obtained by the definition based on d-ROMs levels, the bottom cohort specific TTL quartile (25 %) was used to classify subjects to a high oxidative stress category based on TTL. The cross-sectional analyses were conducted in all subjects and separately for women and men.

**Table 1:** Baseline characteristics of study participants by cohort and case status (death during follow-up) and number of cases for cause-specific mortality analyses by cohort

Baseline characteristics	HAPIEE Poland		HAPIEE Czech Republic		HAPIEE Lithuania		ESTHER Germany	
	Cases (Deaths)	Eligible controls	Cases (Deaths)	Eligible controls	Cases (Deaths)	Eligible controls	Cases (Deaths)	Controls
Total sample size	525	1498	518	1776	390	1278	269	3,758
Age (years)	62.9 (56.7; 67.0)*	62.1 (55.8; 66.5)	63.6 (58.6; 67.4)	63.0 (57.9; 66.8)	66.5 (61.1; 70.3)	66.1 (60.5; 70.1)	74.1 (70.0; 78.1)*	69.2 (64.3; 74.0)
45 to <60	190 (36.2)	594 (39.7)	154 (29.7)	559 (31.5)	79 (20.3)	292 (22.9)	10 (3.7)*	345 (9.2)
60 to <65	126 (24.0)	355 (23.7)	144 (27.8)	518 (29.2)	83 (21.3)	265 (20.7)	18 (6.9)*	681 (18.1)
65 to <70	198 (37.7)	507 (33.9)	198 (38.2)	634 (35.7)	124 (31.8)	391 (30.6)	42 (15.6)*	1020 (27.1)
70 to <85	11 (2.1)	42 (2.8)	22 (4.3)	65 (3.7)	104 (26.7)	330 (25.8)	199 (74.0)*	1712 (45.6)
Male sex	357 (68.0)	1015 (67.8)	339 (65.4)	1170 (65.9)	261 (66.9)	830 (65.0)	158 (58.7)*	1636 (43.5)
Education								
Low	90 (17.2)*	168 (11.2)	90 (17.5)*	175 (10.0)	68 (17.8)*	130 (10.3)	192 (73.3)	2633 (71.1)
Medium	322 (61.5)*	842 (56.2)	377 (73.5)*	1296 (73.7)	181 (47.4)*	463 (36.6)	59 (22.5)	852 (23.0)
High	112 (21.4)*	488 (32.5)	46 (9.0)*	287 (16.3)	133 (34.8)*	672 (53.1)	11 (4.2)	218 (5.9)
BMI (kg/m^2^)	27.4 (24.5; 30.9)	27.9 (25.1; 30.7)	28.7 (25.7; 32.0)*	28.0 (25.5; 30.9)	28.5 (25.4; 32.8)	29.0 (25.7; 32.1)	28.3 (24.9; 31.2)	27.8 (25.1; 30.9)
<20	13 (2.5)	24 (1.6)	10 (1.9)*	20 (1.1)	11 (2.8)*	10 (0.8)	5 (2.0)	47 (1.3)
20 to <25	130 (24.9)	330 (22.1)	92 (17.8)*	344 (19.4)	74 (19.0)*	236 (18.5)	60 (23.4)	857 (23.2)
25 to <30	222 (42.5)	682 (45.6)	207 (40.0)*	827 (46.6)	154 (39.5)*	516 (40.4)	112 (43.6)	1625 (44.0)
30 to <35	110 (21.1)	362 (24.2)	149 (28.8)*	450 (25.4)	98 (25.1)*	369 (28.9)	52 (20.2)	859 (23.3)
≥35	47 (9.0)	98 (6.6)	60 (11.6)*	134 (7.6)	53 (13.6)*	147 (11.5)	28 (10.9)	304 (8.2)
Smoking								
Never	124 (23.8)*	598 (40.0)	140 (27.5)*	761 (43.6)	176 (46.2) *	742 (58.7)	100 (38.8)*	2119 (57.3)
Former	175 (33.5)*	510 (34.1)	192 (37.6)*	598 (34.3)	99 (26.0) *	295 (23.3)	123 (47.7)*	1310 (35.4)
Current	223 (42.7)*	386 (25.8)	178 (34.9)*	386 (22.1)	106 (27.8) *	227 (18.0)	35 (13.6)*	271 (7.3)
Alcohol consumption (g/day)	0 (0; 11.4)*	0 (0; 25.4)	11.4 (2.7; 31.9)*	14.3 (5.7; 30.3)	0 (0; 8)	1.4 (0; 10.3)	3.4 (0; 13.5)*	6.7 (0; 14.4)
Vigorous physical activity	331 (67.8)*	1078 (75.4)	298 (60.8)*	1295 (74.7)	176 (46.4)*	742 (58.7)	87 (41.6)*	2067 (62.4)
Total cholesterol (mg/dL)	218 (192; 251)	219 (195; 247)	213 (186; 239)*	218 (194; 247)	220 (190; 251)*	225 (200; 255)	211 (180; 249)*	233 (202; 265)
<200	180 (34.3)	443 (29.7)	206 (39.9)*	550 (31.0)	124 (31.8)*	323 (25.4)	102 (38.1)*	868 (23.1)
200 to <280	297 (56.6)	930 (62.1)	272 (52.6)*	1088 (61.3)	230 (59.0)*	801 (62.9)	135 (50.4)*	2278 (60.7)
≥280	48 (9.1)	125 (8.3)	39 (7.5)*	138 (7.8)	36 (9.2)*	149 (11.7)	31 (11.6)*	609 (16.2)
HDL cholesterol (mg/dL)	51 (42; 61)*	52 (44; 61)	48 (40; 58)*	49 (41; 60)	53 (43; 63)*	54 (46; 65)	54 (45; 64)*	59 (49; 70)
<40	97 (18.5)*	187 (12.5)	132 (25.5)	369 (20.9)	72 (19.0)*	133 (10.6)	38 (14.2)*	221 (5.9)
40 to <80	392 (74.7)*	1237 (82.6)	359 (69.4)	1313 (74.3)	280 (73.7)*	1024 (81.7)	207 (77.2)*	3098 (82.5)
≥80	36 (6.9)*	74 (4.9)	26 (5.0)	86 (4.9)	28 (7.4)*	96 (7.7)	23 (8.6)*	436 (11.6)
CRP (mg/L)	2.6 (1.1; 5.6)*	1.7 (0.8; 3.1)	2.7 (1.12; 5.6)*	1.9 (0.9; 3.6)	2.7 (1.2; 5.6)*	1.6 (0.8; 3.2)	2.8 (1.3; 6.2)*	1.8 (0.9; 3.7)
≤3	294 (56.0)*	1100 (73.4)	281 (54.3)*	1218 (68.6)	214 (54.9)*	931 (72.9)	143 (53.2)*	2567 (68.3)
>3 to ≤10	165 (31.4)*	328 (21.9)	185 (35.7)*	474 (26.7)	125 (32.1)*	276 (21.6)	96 (35.7)*	967 (25.7)
>10	66 (12.6)*	70 (4.7)	52 (10.0)*	84 (4.7)	51 (13.1)*	71 (5.6)	30 (11.2)*	224 (6.0)
eGFR (mL/min/1.73 m^2^)	89.2 (76.1; 97.5)	88.6 (77.7; 95.7)	78.2 (64.9; 90.4)*	80.1 (69.3; 90.6)	85.0 (70.0; 93.4)	86.5 (74.1; 94.2)	63.9 (51.8; 76.9)*	71.8 (62.7; 80.7)
≥60	487 (92.7)*	1440 (96.1)	426 (82.2)*	1600 (90.1)	336 (86.2)*	1184 (92.6)	163 (60.6)*	3001 (80.0)
<60	38 (7.2)*	58 (3.9)	92 (17.8)*	176 (9.9)	54 (13.9)*	94 (7.4)	106 (39.4)*	757 (20.1)
History of hypertension	323 (61.9)*	837 (55.9)	300 (58.6)*	902 (50.9)	248 (65.1)	788 (62.4)	221 (82.2)*	2637 (70.2)
History of diabetes	111 (21.2)*	207 (13.8)	123 (24.0)*	243 (13.7)	56 (14.7)*	91 (7.2)	108 (40.2)*	892 (23.7)
History of MI	110 (21.1)*	147 (9.9)	69 (14.1)*	104 (6.1)	71 (18.6)*	121 (9.6)	50 (18.6)*	217 (5.8)
History of stroke	31 (6.0)*	33 (2.2)	40 (8.2)*	59 (3.5)	35 (9.2)*	68 (5.4)	46 (17.1)*	262 (7.0)
History of cancer	52 (9.9)*	67 (4.5)	53 (10.2)*	93 (5.2)	58 (14.9)*	104 (8.1)	94 (34.9)*	428 (11.4)
d-ROMs (Carr U)	368 (324; 425)*	349 (309; 394)	404 (350; 470)*	381 (335; 439)	372 (330; 421)*	356 (317; 393)	369 (320; 406)*	350 (305; 395)
≤340	178 (33.9)*	650 (43.4)	102 (19.7)*	483 (27.2)	123 (31.5)*	498 (39.0)	96 (35.7)*	1649 (43.9)
341–400	167 (31.8)*	514 (34.1)	146 (28.2)*	564 (31.8)	138 (35.4)*	500 (39.1)	94 (34.9)*	1250 (33.3)
401–500	143 (27.2)*	314 (21.0)	185 (35.7)*	561 (31.6)	101 (25.9)*	258 (20.2)	70 (26.0)*	789 (21.0)
>500	37 (7.0)*	20 (1.3)	85 (16.4)*	168 (9.5)	28 (7.2)*	22 (1.7)	9 (3.3)*	70 (1.9)
TTL (μmol/L)	509 (445; 566)*	518 (464; 570)	406 (363; 452)	411 (371; 450)	313 (273; 357)*	321 (283; 359)	303 (239; 356) *	335 (284; 383)
Cause of death								
CVD	165 (32.0)	n.a.	189 (36.6)	n.a.	155 (39.7)	n.a.	51 (31.7)	n.a.
Cancer	235 (45.5)	n.a.	235 (45.5)	n.a.	158 (40.5)	n.a.	74 (46.0)	n.a.
Non-CVD, non-cancer	116 (22.5)	n.a.	93 (18.0)	n.a.	77 (19.7)	n.a.	36 (22.4)	n.a.
Unknown	9 (n.a.)	n.a.	1 (n.a.)	n.a.	0 (n.a.)	n.a.	108 (n.a.)^a^	n.a.

Unless indicated otherwise, the table shows proportions (%) for categorical and medians (25^th^; 75^th^ percentile) for continuous variables. Numbers shown were drawn from the not imputed data set. Therefore, numbers do not always add up to the total because of missing values (see Additional file 1: Table S1 for proportion of missing values for each variable)

*Statistically significant (*P* <0.05) difference among cases and controls assessed by χ^2^ test for categorical and Wilcoxon rank sum test for continuous variables

BMI, Body mass index; CRP, C-reactive protein; CVD, Cardiovascular disease; d-ROMs, Derivatives of reactive oxygen metabolites; eGFR, Estimated glomerular filtration rate; HDL, High-density lipoprotein; MI, Myocardial infarction; n.a., Not applicable; TTL, Total thiol levels

^a^Information on cause of death is released by German authorities later than general mortality information. At the time of the analysis, information about all-cause mortality was available until July 31, 2014, and information about causes of deaths for deaths that occurred until April 2, 2013

For longitudinal analyses, Cox proportional hazard regression (ESTHER cohort) and conditional logistic regression (HAPIEE matched case–control study) were utilized to estimate hazard ratios and odds ratios, respectively, for an increase in d-ROMs levels and decrease in TTL by one cohort-specific standard deviation (SD). In addition, d-ROMs levels were also modelled as a categorical variable, with manufacturer-recommended clinical cut-offs for moderate (341–400 Carr U), high (401–500 Carr U) and very high oxidative stress (>500 Carr U) with reference to subjects with not increased or low oxidative stress (≤340 Carr U). When the absolute risk is low, logistic and Cox regression usually produce similar results and the odds ratio can be regarded as an approximation of the more accurate hazard ratio. We used the term relative risk (RR) for both effect estimates. In sensitivity analysis, analyses on cause-specific mortality in the ESTHER cohort were carried out considering the competing risk of death due to other causes by fitting a proportional subdistribution hazards regression model with weights for subjects that underwent the competing risk event according to an extension of the Fine and Gray method [21]. However, results were almost identical with traditional Cox proportional hazard regression and therefore only the latter results are shown in this manuscript.

Analyses were conducted separately for each cohort and pooled by meta-analysis with Mantel-Haenszel weighting and random effects [22], taking the sample size of the cohorts and the possibility of statistical heterogeneity among the studies into account. The latter was examined with Cochrane’s Q test and the I^2^ statistic.

The following outcomes were analysed: mortality from all-causes, CVD, cancer and non-CVD, non-cancer causes. For each outcome, four statistical models were developed, with an increasing inclusion of established mortality risk factors into the models. Both d-ROMs levels and TTL were always included in the same model because their correlation was low (r <0.08 in each cohort). In model 1, adjustment was made for age and sex in the ESTHER cohort, whereas this was not necessary for the HAPIEE cohorts because cases were individually matched to two controls by age and sex and analysed in stratified analysis in which the strata consist of the collection of matched sets. Model 2 additionally adjusted for education, body mass index (BMI), smoking, alcohol consumption and physical activity. Model 3 also included diseases that could potentially mediate the association of oxidative stress with mortality (i.e. dyslipidaemia (assessed by total and HDL cholesterol), renal impairment, a history of diabetes, hypertension, MI, stroke, and cancer). Finally, model 4 was additionally adjusted for CRP levels, which were strongly correlated with d-ROMs levels (r = 0.34–0.41 in the cohorts) and, less strongly, with TTL (r = 0.11–0.17 in the cohorts). Age was modelled as a continuous variable and all other variables were modelled as categorical variables (categories are shown in Table 1). We tested for interactions between d-ROMs/TTL with covariates by adding appropriate interaction terms to model 2.

Subgroups to be analysed were chosen a priori with the aim to stratify for the most important potential determinants of high oxidative stress levels: cohort/country, age, sex, history of MI or cancer, and inflammatory status. Because of sample size limitations, stratified analyses were restricted to mortality of all causes, CVD, and cancer. To address the possibility of reverse causation bias [23], analyses were conducted separately for events that occurred in years 1–2, years 3–4, and years 5–6 of follow-up. Because of sample size limitations, this stratified analysis could only be performed for all-cause mortality. To avoid model instability, a cohort-specific subgroup analysis result was only considered for meta-analysis when at least 25 events occurred in the sub-group.

Multiple imputation was employed to impute the number of missing baseline covariate values shown in Additional file 1: Table S1. The proportion of missing values was below 5 % for all variables with the exception of alcohol consumption and physical activity, which had up to 22 % of missing values. To the best of our knowledge, data were missing at random, which is the assumption of the multiple imputation. Separately by cohort, case status and sex, 20 complete data sets were imputed with the SAS 9.3 procedure “PROC MI”, using the Markov chain Monte Carlo method. Variables from the “full” model were used for the imputation model. All multivariable analyses were performed in the 20 imputed data sets and results of the individual data sets were combined by the SAS 9.3 procedure “PROC MIANALYZE”.

The meta-analyses were conducted with the statistical software Comprehensive Meta-Analysis 2.0 (Biostat, Englewood, NJ, USA). All other analyses were conducted with SAS, version 9.2 (Cary, North Carolina, USA). All statistical tests were two-sided, using an alpha level of 0.05.

## Results

### Comparison of baseline characteristics of the analysed cohorts and of cases and controls

Baseline characteristics of cases and controls in the four cohorts are shown in Table 1. The HAPIEE cohorts included approximately two thirds of men and one third of women among cases and controls (because of the sampling, see Additional file 1: Figure S1), whereas more men were among the cases and more women among the controls in the ESTHER study. Because of the sampling of subjects in the HAPIEE cohorts, the age distribution among cases and controls was comparable, whereas cases were much older than controls in the ESTHER study. Controls from the ESTHER study were on average 7.1, 6.2, and 3.1 years older than controls from the HAPIEE Poland, Czech Republic, and Lithuania studies, respectively. Comparing only cases with cases and controls with controls in the different cohorts, most baseline characteristics were comparably distributed among the cohorts, with few exceptions that could mostly be explained by the age differences of the cohorts. Differences observed for years of school education could be explained by the different educational systems of the countries and different years of school enrolment of study participants. The higher prevalence of diabetes in the ESTHER study is due to the fact that ESTHER study participants were screened for undetected diabetes at baseline, which was not done in the HAPIEE cohorts. With regards to the oxidative stress markers, median d-ROMs levels were similar in HAPIEE Lithuania, HAPIEE Poland and ESTHER, and higher in HAPIEE Czech Republic than in the other three cohorts. Differences in TTL were much more pronounced than those of d-ROMs levels and mirror the age differences of the cohorts. The causes of deaths were similarly distributed among cases from the cohorts, with roughly 35 % being CVD deaths, 45 % cancer deaths, and 20 % deaths from other causes. All the assessed established risk factors for mortality were found to be statistically significantly associated with this outcome in the majority of the analysed studies (Table 1).

### Cross-sectional associations of established risk factors for mortality and high oxidative stress levels

Associations between high oxidative stress levels (defined as d-ROMs levels >400 Carr U or bottom cohort-specific TTL quartile) were similar in the ESTHER cohort, HAPIEE cases, and HAPIEE controls, with low heterogeneity between studies (Additional file 1: Tables S2-S3). In the meta-analyses, female sex and increasing CRP levels were associated with higher odds for both definitions of high oxidative stress (Table 2), whereas alcohol consumption, physical activity, and a history of diabetes or stroke were not associated with both definitions. However, high oxidative stress defined by d-ROMs was associated with smoking, increasing total cholesterol, increasing HDL cholesterol, and a history of hypertension, MI or cancer, whereas high oxidative stress by TTL was not associated with these factors. Conversely, high oxidative stress defined by TTL was associated with increasing age, increasing education, increasing BMI, and renal impairment, whereas high oxidative stress defined by d-ROMs levels was not associated with these factors. The unexpected direction of the association of BMI and increased d-ROMs levels was introduced by one outlier (cases of HAPIEE Lithuania study) and adjustment for diseases and CRP. In the meta-analysis of results from the main model 2 and exclusion of cases from HAPIEE Lithuania, overweight and adiposity grade I (BMI, 30 to <35 kg/m^2^) were not associated with high oxidative stress defined by d-ROMs levels and adiposity grade II–III (BMI, ≥35 kg/m^2^) was statistically significantly associated with increased d-ROMs (Additional file 1: Table S4), i.e. the same direction as observed for high oxidative stress defined by TTL. Similarly, the other paradox directions of risk factors with oxidative stress (increasing HDL with high oxidative stress by d- ROMs and increasing education with high oxidative stress by TTL) were not observed in models that were only adjusted for age and sex (Additional file 1: Tables S4,S5).

**Table 2:** Cross-sectional associations of established risk factors for mortality and high oxidative stress levels defined by derivatives of reactive oxygen metabolites (d-ROMs) levels or total thiol levels (TTL)

Risk factors for mortality	Odds of high oxidative stress by	
	d-ROMs^a^	TTL^b^
Increasing age	–	↑
Male sex	↓	↓
Decreasing education	–	(↓)^c^
Increasing BMI	(↓)^d^	↑
Former smoking	↑	–
Current smoking	↑	–
No alcohol consumption	–	–
High alcohol consumption^e^	–	–
No performance of vigorous physical activity	–	–
Increasing total cholesterol	↑	–
Decreasing HDL cholesterol	(↓)^f^	–
Increasing CRP	↑	↑
Renal impairment^g^	–	↑
History of hypertension	↑	–
History of diabetes	–	–
History of MI	↑	–
History of stroke	–	–
History of cancer	↑	–

^a^d-ROMs >400 Carr U

^b^Lowest quartile of TTL in the different cohorts, i.e. ESTHER <281.57 μmol/L; HAPIEE Poland cases and eligible controls <464 μmol/L; HAPIEE Czech Republic cases and eligible controls <371 μmol/L; HAPIEE Lithuania cases and eligible controls <283 μmol/L

^c^No association in age- and sex-adjusted model (Additional file 1: Table S4)

^d^No association in main model for all BMI categories with exception of the highest BMI category, which showed a statistically significantly increased odds for high oxidative stress (Additional file 1: Table S3)

^e^Definition of high alcohol consumption: women ≥20 and men ≥40 g ethanol per day

^f^No association in main model (Additional file 1: Table S3)

^g^Estimated glomerular filtration rate <60 mL/min/1.73 m^2^

BMI, Body mass index; CRP, C-reactive protein; HDL, High-density lipoprotein; MI, Myocardial infarction; d-ROMs, Derivatives of reactive oxygen metabolites; TTL, Total thiol levels

A strongly decreased odds of males compared to females for high oxidative stress by both definitions was observed in all models and was stronger for d-ROMs levels than TTL (Additional file 1: Table S6). However, the cross-sectional associations of baseline characteristics with high oxidative stress were similar in men and women (Additional file 1: Tables S7,S8). Consistent sex differences were found only for current smoking and CRP, which showed a stronger association with high oxidative stress levels (defined by d-ROMs levels) in men than in women (Additional file 1: Table S7) and for high age (70–85 years), which was more strongly associated with high oxidative stress levels (defined by TTL) in women than in men (Additional file 1: Table S8).

### Longitudinal association of oxidative stress markers with mortality outcomes

The HAPIEE Poland, Czech Republic, Lithuania, and ESTHER cohorts were followed-up for mortality for a median of 7.1, 8.1, 4.6, and 5.0 years, respectively. During the follow-up, 525, 518, 390, and 269 study participants in the respective cohorts died. From the 1,702 deaths in total, 560 were of cardiovascular and 702 of cancer origin.

d-ROMs levels and TTL were statistically significantly associated with all-cause mortality in all models, with stronger effect estimates for d-ROMs levels than for TTL (Table 3). Although the strength of the associations declined with increasing adjustment for other factors, a 1.3-fold increased mortality for high oxidative stress levels (d-ROMs levels: 401–500 Carr U) and a 2.3-fold increased mortality for very high oxidative stress levels (d-ROMs levels >500 Carr U) remained statistically significant in the fully adjusted model 4. Compared to the results for all-cause mortality, results for d-ROMs levels were stronger for CVD mortality, similar for cancer mortality and weaker for non-CVD, non-cancer mortality. Effect estimates for a decrease in TTL by one SD with CVD mortality and non-CVD, non-cancer mortality were comparable to the effect estimates observed for an increase of d-ROMs levels by one SD. However, TTL were not associated with cancer mortality in any model and not statistically significantly associated with non-CVD, non-cancer mortality in models 3 and 4.

**Table 3:** Associations of derivatives of reactive oxygen metabolites (d-ROMs) levels and total thiol levels (TTL) with all-cause and disease-specific mortality

Outcome	Oxidative stress marker	Modelling	n_cases_	Model 1	Model 2	Model 3	Model 4
				RR (95 % CI)^a^	RR (95 % CI)^b^	RR (95 % CI)^c^	RR (95 % CI)^d^
All-cause mortality	d-ROMs	≤340 Carr U	499	Ref	Ref	Ref	Ref
		341–400 Carr U	545	1.27 (1.07–1.51)*	1.21 (1.04–1.40)*	1.20 (1.03–1.40)*	1.13 (0.96–1.32)
		401–500 Carr U	499	1.86 (1.58–2.17)*	1.60 (1.35–1.89)*	1.53 (1.29–1.83)*	1.32 (1.10–1.59)*
		>500 Carr U	159	4.48 (2.87–7.00)*	3.79 (2.08–6.90)*	2.99 (1.62–5.52)*	2.30 (1.40–3.77)*
	d-ROMs	Increase per 1 SD^e^	1702	1.44 (1.35–1.53)*	1.34 (1.25–1.43)*	1.28 (1.19–1.36)*	1.20 (1.12–1.29)*
	TTL	Decrease per 1 SD^f^	1702	1.17 (1.04–1.33)*	1.19 (1.07–1.32)*	1.14 (1.06–1.23)*	1.12 (1.04–1.21)*
CVD mortality	d-ROMs	≤340 Carr U	157	Ref	Ref	Ref	Ref
		341–400 Carr U	178	1.29 (1.00–1.67)*	1.28 (0.97–1.70)	1.28 (0.89–1.82)	1.14 (0.83–1.56)
		401–500 Carr U	171	1.71 (1.32–2.22)*	1.78 (1.31–2.43)*	1.81 (1.28–2.54)*	1.49 (1.04–2.13)*
		>500 Carr U^g^	54	5.16 (2.35–11.31)*	5.09 (2.67–9.69)*	4.86 (2.38–9.95)*	4.34 (2.06–9.15)*
	d-ROMs	Increase per 1 SD^e^	560	1.53 (1.35–1.72)*	1.44 (1.27–1.64)*	1.37 (1.19–1.58)*	1.30 (1.12–1.51)*
	TTL	Decrease per 1 SD^f^	560	1.31 (1.17–1.47)*	1.30 (1.15–1.48)*	1.27 (1.10–1.47)*	1.25 (1.09–1.45)*
Cancer mortality	d-ROMs	≤340 Carr U	198	Ref	Ref	Ref	Ref
		341–400 Carr U	224	1.27 (1.01–1.61)*	1.18 (0.92–1.51)	1.17 (0.91–1.52)	1.08 (0.83–1.41)
		401–500 Carr U	200	1.71 (1.32–2.21)*	1.48 (1.12–2.75)*	1.41 (1.05–1.89)*	1.16 (0.85–1.57)
		>500 Carr U	80	5.29 (3.16–8.85)*	4.34 (2.31–8.16)*	3.82 (1.79–8.18)*	2.50 (1.31–4.78)*
	d-ROMs	Increase per 1 SD^e^	702	1.42 (1.29–1.56)*	1.35 (1.20–1.52)*	1.30 (1.14–1.47)*	1.19 (1.05–1.35)*
	TTL	Decrease per 1 SD^f^	702	1.05 (0.92–1.19)	1.07 (0.96–1.19)	1.01 (0.90–1.13)	0.98 (0.87–1.09)
Non-CVD, non-cancer mortality	d-ROMs	≤340 Carr U	103	Ref	Ref	Ref	Ref
		341–400 Carr U	99	1.15 (0.80–1.67)	0.94 (0.52–1.68)	0.97 (0.51–1.82)	0.85 (0.48–1.49)
		401–500 Carr U	99	2.14 (1.51–3.05)*	1.49 (0.91–2.45)	1.51 (0.89–2.55)	1.16 (0.67–2.01)
		>500 Carr U	21	3.32 (1.68–6.59)*	1.98 (0.94–4.15)	1.93 (0.83–4.45)	1.56 (0.69–3.53)
	d-ROMs	Increase per 1 SD^e^	322	1.41 (1.22–1.63)*	1.26 (1.08–1.47)*	1.27 (1.07–1.51)*	1.15 (0.96–1.37)
	TTL	Decrease per 1 SD^f^	322	1.23 (1.07–1.42)*	1.27 (1.02–1.60)*	1.19 (0.94–1.48)	1.15 (0.93–1.43)

*Statistically significant (*P* <0.05)

^a^Adjusted for age and sex in the ESTHER cohort, whereas this was not necessary for the HAPIEE cohorts because cases were individually matched to two controls by age and sex and analysed in stratified analysis in which the strata consist of the collection of matched sets. In addition, the other oxidative stress marker (i.e. TTL or d-ROMs) was included in this model

^b^Variables of model 1 and education, BMI, smoking, alcohol consumption, and vigorous physical activity

^c^ Variables of model 2 and total cholesterol, HDL cholesterol, renal impairment, history of diabetes, history of hypertension, history of myocardial infarction, history of stroke, and history of cancer

^d^Variables of model 3 and CRP

^e^1 SD of d-ROMs levels equal 81.1, 89.6, 70.5, and 71.9 Carr U in the HAPIEE Poland, Czech Republic, Lithuania, and ESTHER studies, respectively

^f^1 SD of TTL equals 79.5, 69.1, 74.1, and 75.3 μmol/L in the HAPIEE Poland, Czech Republic, Lithuania, and ESTHER studies, respectively

^g^Meta-analyses without the ESTHER study because no events were observed in this category in this study

CVD, Cardiovascular disease; CI, Confidence interval; d-ROMs, Derivatives of reactive oxygen metabolites; n_cases_, Incident case numbers; Ref, Reference category; SD, Standard deviation; TTL, Total thiol levels

### Additive risks by d-ROMs levels and TTL

No statistically significant interactions were observed between d-ROMs levels and TTL and between the two oxidative stress markers and other risk factors. However, d-ROMs levels and TTL had additive predictive values for CVD mortality (Table 4). Subjects with high oxidative stress by both definitions had the highest RR for CVD mortality (RR, 2.47; 95 % CI, 1.58–3.98) with about twice the excess risk of subjects with high oxidative stress defined by only d-ROMs levels (147 % excess risk compared to 72 % excess risk). Agreement of the two definitions of high oxidative stress was relatively low and only 7 % of the ESTHER population and 9 % of eligible controls of the HAPIEE cohorts fulfilled both definitions.

**Table 4:** Joint risk assessment for cardiovascular disease mortality by derivatives of reactive oxygen metabolites (d-ROMs) levels and total thiol levels (TTL)

High oxidative stress by		Proportion of population		n_cases_	RR (95 % CI)^c^
d-ROMs^a^	TTL^b^	ESTHER	HAPIEE controls	ESTHER + HAPIEE	ESTHER + HAPIEE
No	No	59 %	49 %	227	Ref
No	Yes	18 %	25 %	108	1.34 (0.91–1.97)
Yes	No	16 %	18 %	155	1.72 (1.27–2.32)*
Yes	Yes	7 %	9 %	70	2.47 (1.58–3.98)*

*Statistically significant (*P* <0.05)

^a^d-ROMs levels >400 Carr U

^b^Lowest quartile of TTL in the different cohorts, i.e. ESTHER <281.57 μmol/L; HAPIEE Poland cases and eligible controls <464 μmol/L; HAPIEE Czech Republic cases and eligible controls <371 μmol/L; HAPIEE Lithuania cases and eligible controls <283 μmol/L

^c^Meta-analysis of HAPIEE Poland, Czech Republic, Lithuania and ESTHER study. Model controlled for age, sex, education, BMI, smoking status, alcohol consumption and vigorous physical activity

CI, Confidence interval; n_cases_, Incident case numbers; RR, Risk ratio

### Subgroup analyses

Table 5 shows the associations of TTL and d-ROMs levels with all-cause, CVD and cancer mortality stratified by cohort/country, age, sex, history of MI or cancer, inflammatory status and time-point of event during follow-up. With respect to cohort/country, results for d-ROMs levels were homogenous and statistically significant in all cohorts/countries, with few exceptions (non-significant association with CVD mortality in ESTHER and with cancer mortality in HAPIEE Poland). For TTL, there was a tendency towards stronger effect estimates in the older cohorts (HAPIEE Lithuania and ESTHER) than in the younger cohorts (HAPIEE Poland and Czech Republic). This age difference for TTL was also observed when analyses were stratified by age groups. The strongest effect estimates for all-cause and CVD mortality were observed in the oldest age group (70–85 years). Conversely, there was no clear age trend for d-ROMs. However, d-ROMs levels were not statistically significantly associated with CVD mortality in the oldest age group and not with cancer mortality in the youngest age group (45 to <60 years).

**Table 5:** Association of derivatives of reactive oxygen metabolites (d-ROMs) levels and total thiol levels (TTL) with all-cause and disease specific mortality stratified by cohort/country, sex, age, history of myocardial infarction, history of cancer, inflammatory status and time-point of event during follow-up

Outcome	Stratum	n_cases_	Increase of d-ROMs by 1 SD^a^	Decrease of TTL by 1 SD^b^
			RR (95 % CI)^c^	RR (95 % CI)^c^
Stratified by cohort/country				
All-cause mortality	HAPIEE Poland	525	1.29 (1.14–1.46)*	1.14 (1.01–1.28)*
	HAPIEE Czech Republic	518	1.33 (1.17–1.51)*	1.05 (0.93–1.19)
	HAPIEE Lithuania	390	1.47 (1.25–1.71)*	1.31 (1.10–1.57)*
	ESTHER (Germany)	269	1.33 (1.18–1.49)*	1.31 (1.15–1.48)*
CVD mortality	HAPIEE Poland	165	1.54 (1.21–1.95)*	1.22 (0.98–1.53)
	HAPIEE Czech Republic	189	1.56 (1.22–2.00)*	1.25 (0.99–1.56)
	HAPIEE Lithuania	155	1.40 (1.06–1.85)*	1.48 (1.06–2.06)*
	ESTHER (Germany)	51	1.24 (0.94–1.63)	1.39 (1.06–1.84)*
Cancer mortality	HAPIEE Poland	235	1.17 (0.98–1.41)	1.06 (0.89–1.26)
	HAPIEE Czech Republic	235	1.36 (1.13–1.63)*	0.95 (0.79–1.14)
	HAPIEE Lithuania	158	1.60 (1.25–2.04)*	1.18 (0.89–1.55)
	ESTHER (Germany)	74	1.40 (1.13–1.74)*	1.21 (0.96–1.53)
Stratified by age				
All-cause mortality	45 to <60 years^d^	423	1.23 (1.04–1.45)*	1.16 (1.01–1.34)*
	60 to <70 years	933	1.39 (1.27–1.53)*	1.16 (1.05–1.29)*
	70 to <85 years^e, f^	303	1.33 (1.17–1.52)*	1.35 (1.18–1.55)*
CVD mortality	45 to <60 years^d^	126	1.42 (1.01–2.00)*	1.24 (0.91–1.69)
	60 to <70 years^d^	322	1.64 (1.35–2.00)*	1.31 (1.09–1.56)*
	70 to <85 years^e, f^	90	1.28 (0.97–1.69)	1.65 (1.22–2.24)*
Cancer mortality	45 to <60 years^d^	177	1.21 (0.81–1.81)	1.20 (0.96–1.50)
	60 to <70 years^d^	392	1.41 (1.21–1.64)*	1.02 (0.86–1.21)
	70 to <85 years^e, f^	94	1.46 (1.13–1.87)*	1.17 (0.89–1.52)
Stratified by sex				
All-cause mortality	Women	587	1.28 (1.13–1.44)*	1.25 (1.05–1.50)*
	Men	1115	1.40 (1.29–1.53)*	1.18 (1.08–1.28)*
CVD mortality	Women^d^	157	1.42 (1.08–1.87)*	1.28 (0.98–1.69)
	Men	385	1.50 (1.27–1.76)*	1.33 (1.13–1.56)*
Cancer mortality	Women	257	1.31 (1.05–1.65)*	1.16 (0.96–1.40)
	Men	445	1.39 (1.21–1.59)*	1.06 (0.93–1.21)
Stratified by history of MI				
All-cause mortality	No history of MI	1286	1.33 (1.22–1.41)*	1.17 (1.04–2.61)*
	History of MI^g^	375	1.39 (1.17;1.65 )*	1.19 (0.97–1.63)
CVD mortality	No history of MI	379	1.35 (1.14–1.57)*	1.22 (1.04–1.43)*
	History of MI^d, g^	165	1.45 (1.04–2.02)*	1.19 (0.88–1.61)
Stratified by history of cancer				
All-cause mortality	No history of cancer	1382	1.32 (1.23–1.42)*	1.16 (1.05–1.29)*
	History of cancer^g^	257	1.49 (1.24–1.77)*	1.24 (1.00–1.54)*
Cancer mortality	No history of cancer	533	1.29 (1.15–1.45)*	1.01 (0.89–1.14)
	History of cancer^g^	169	1.53 (1.19–1.97)*	1.16 (0.91–1.47)
Stratified by inflammatory status				
All-cause mortality	CRP ≤3 mg/L	933	1.19 (1.08–1.31)*	1.12 (0.97–1.30)
	CRP >3 mg/L	769	1.26 (1.15–1.38)*	1.16 (1.05–1.28)*
CVD mortality	CRP ≤3 mg/L^d^	265	1.26 (1.00–1.58)*	1.10 (0.90–1.35)
	CRP >3 mg/L	275	1.28 (1.07–1.54)*	1.22 (1.01–1.48)*
Cancer mortality	CRP ≤3 mg/L	394	1.22 (1.04–1.43)*	1.06 (0.90–1.26)
	CRP >3 mg/L	308	1.27 (1.10–1.46)*	1.07 (0.91–1.26)
Stratified by time-point of event during follow-up				
All-cause mortality	≤ year 2	419	1.46 (1.29–1.66)*	1.29 (1.11–1.50)*
	> year 2 to ≤ year 4	597	1.31 (1.17–1.46)*	1.18 (1.06–1.33)*
	> year 4 to ≤ year 6	433	1.25 (1.09–1.43)*	1.17 (1.02–1.33)*

*Statistically significant (*P* <0.05)

^a^1 SD of d-ROMs levels equal to 81.1, 89.6, 70.5, and 71.9 Carr U in the HAPIEE Poland, Czech Republic, Lithuania, and ESTHER study, respectively

^b^1 SD of TTL equals 79.5, 69.1, 74.1, and 75.3 μmol/L in the HAPIEE Poland, Czech Republic, Lithuania, and ESTHER study, respectively

^c^Meta-analysis of HAPIEE Poland, Czech Republic, Lithuania, and ESTHER study if not stated otherwise. Model controlled for age, sex, education, BMI, smoking status, alcohol consumption, vigorous physical activity, and the other oxidative stress marker (i.e. TTL or d-ROMs)

^d^Meta-analyses without the ESTHER study because too few events (<25) were observed in this category in this study

^e^Meta-analyses without the HAPIEE Poland study because too few events (<25) were observed in this category in this study

^f^ Meta-analyses without the HAPIEE Czech Republic study because too few events (<25) were observed in this category in this study

^g^One instead of two matched controls due to a shortage of potential controls in this category in the HAPIEE cohorts

CVD, Cardiovascular disease; CI, Confidence interval; CRP, C-reactive protein; MI, Myocardial infarction; n_cases_, Incident case numbers; RR, Risk ratio; SD, Standard deviation

We did not find strong sex differences, but the associations of d-ROMs levels with mortality outcomes had a tendency towards slightly stronger results in men than in women. Furthermore, there was a tendency towards stronger results for d-ROMs levels in subjects with a history of MI or with a history of cancer but effect estimates were also statistically significant in subjects without a history of these diseases. Results for TTL showed no relevant differences in diseased and non-diseased study participants. With regards to the inflammatory status, subjects with and without subclinical inflammation (CRP ≤3 vs. >3 mg/L) had almost identically strong associations of d-ROMs levels with all-cause, CVD and cancer mortality, but the association of TTL with all-cause and CVD mortality was significant only in subjects with subclinical inflammation. Finally, although the associations of d-ROMs levels and TTL were weaker with events occurring later in the follow-up, even associations with events that occurred 4–6 years after baseline were statistically significant in the meta-analysis of the four cohorts. All heterogeneity statistics for meta-analyses of longitudinal analyses of the included four cohorts indicated low, statistically non-significant levels of heterogeneity.

## Discussion

### Principal findings

In this meta-analysis of four population-based cohort studies from Central and Eastern Europe, the serum oxidative stress markers d-ROMs and TTL were associated with all-cause mortality independently from each other and independently from other risk factors for mortality, including inflammation. d-ROMs levels were strongly associated with all major causes of death, whereas TTL were only associated with CVD mortality. The strength of the association of TTL with CVD mortality increased with age of the participants and was additive to the excess risk of d-ROMs levels. Especially, individuals with very high d-ROMs levels seem to have a particularly high risk for short-term mortality (corresponding to 4- to 5-fold increased mortality from CVD and cancer).

### Strength and weaknesses

This meta-analysis of individual participant data has several strengths, including the variety of cohorts from Central and Eastern Europe, the broad age range from 45–84 years, the overall large size enabling subgroup and cause-specific mortality analyses, almost complete registry-based mortality follow-ups, the common statistical analysis strategy, and the detailed assessment of all major risk factors for mortality enabling a comprehensive correction for confounding.

However, due to the observational nature of the study, confounding cannot be totally excluded and any causal inference has to be made with caution. For example, in order to adjust for inflammation, we used the general inflammatory biomarker CRP, whereas a set of more specific inflammatory markers (e.g. interleukins) could have further reduced any potential confounding by inflammation. Furthermore, the study results cannot be generalized to non-Caucasians. A further limitation is that we did not use repeated d-ROMs and TTL measurements during follow-up which could have led to stronger effect estimates because effect estimates were weaker for events that occurred later during follow-up. However, we were able to show that associations of d-ROMs levels and TTL remained statistically significant with deaths that occurred 5–6 years after baseline, showing their predictive value not only for short-term mortality but also for up to 6-year mortality. Nevertheless, studies with repeated measures and even longer follow-up would be helpful to shed more light on the predictive value of d-ROMs levels and TTL for long-term mortality. Finally, we would like to state that the total thiol/total disulfide ratio would have been a better indicator of the oxidative status than TTL alone. However, disulfides were not measured because, to date, a reliable and time-efficient laboratory method for large-scale measurements is not available.

### Comparison with other studies

This study with up to 8-year follow-up is a major improvement compared to the first analysis of the ESTHER study, with 3.3 years of follow-up [10]. In the current analysis, the ESTHER follow-up was extended to 5 years, and the sample size with d-ROMs and TTL measurements was increased from 2,932 to 4,027 subjects. The increase in statistical power was even larger by the addition of the HAPIEE cohorts with their high case numbers. The results for all-cause mortality were similar to those of the first ESTHER analysis and were corroborated by the results from HAPIEE Poland, Czech Republic, and Lithuania. As in the earlier analysis, we observed attenuations of the effect estimates for TTL and d-ROMs levels after adjustment for history of chronic diseases and CRP but the results remained statistically significant for both TTL and d-ROMs levels, indicating that both markers are risk factors for mortality independent of the presence of common diseases and a high inflammatory status.

With the large numbers of cases in the HAPIEE cohorts, it was possible, for the first time, to address cause-specific mortality. Interestingly, high d-ROMs levels seem to be a general risk factor for all major causes of death, whereas the weaker association of TTL with all-cause mortality seems to be specific to CVD mortality. No other comparable studies have assessed the association of oxidative stress markers with cause-specific mortality in the general population thus far. For all-cause mortality, several population-based cohort studies restricted to older adults (≥65 years) applied other oxidative stress markers and observed significant associations of the oxidative DNA damage marker urinary 8-iso-prostaglandin F_2α_ [24], the anti-oxidant nutrient selenium [25], and the protein oxidation marker protein carbonyls [26] with total mortality. These oxidative stress markers do not measure hydrogen peroxides or free thiol groups, as the d-ROMs and TTL assays, respectively, do. The fact that different assays for the common concept of oxidative stress lead to similar findings provides support for the free radical/oxidative stress theory of ageing. This evidence is further supported by studies with transgenic and knockout mice, which propose oxidative stress biomarkers as risk factors for premature death [5].

### Interpretation of the findings

According to the free radical/oxidative stress theory of ageing, an increased production of ROS promotes cell senescence and an increased apoptosis rate [4, 5]. Oxidative stress occurs when ROS cannot be balanced by anti-oxidative capacities in cells and tissues. In our study, d-ROMs levels and TTL were virtually uncorrelated and were independently associated with CVD mortality. This implies that oxidative and anti-oxidative molecules in blood may influence each other less than one would expect.

Some insights on different mechanisms of action by d-ROMs and TTL can be drawn from their diverging cross-sectional associations with baseline characteristics. The only agreement was found in the fact that female sex and increasing CRP levels were strongly associated with higher odds of both high d-ROMs levels and low TTL. This is as expected since ROS can provoke and potentiate inflammatory processes and vice versa [3]. In addition, previous studies have already reported correlations of oxidative stress biomarkers with female hormones [27]. Besides that, high oxidative stress by d-ROMs levels was associated with smoking, increasing total cholesterol, increasing HDL cholesterol, and a history of hypertension, MI or cancer, whereas high oxidative stress by TTL was associated with increasing age, increasing BMI, and renal impairment. It appears that smoking and major chronic diseases tend to be related to d-ROMs levels, whereas classical factors increasing with age (i.e. BMI, renal impairment, increasing subclinical inflammation) are rather related to TTL. Interestingly, the association of TTL with CVD mortality increased with age of the participants whereas the association of d-ROMs levels with mortality outcomes was independent from participant age.

These results suggest that pro-oxidative processes remain stable in middle and older age (with smokers and subjects with diseases having higher oxidative stress) and can be hazardous throughout the adult life-time (especially because of the association of d-ROMs levels with cancer), whereas anti-oxidative capacities in blood decrease with age (as shown by Pandey et al. [28]), which becomes problematic when they can no longer outbalance pro-oxidative situations at older age (especially after the age of 70 years in our cohorts). It is known that, at the end of life, protein structures essential for protein function and thiol-based redox signaling are increasingly less well maintained, which can contribute to organ function failure and ultimately death of the organism [28, 29]. Moreover, oxidative processes play an important role in the development and rupture of atherosclerotic plaques in blood vessels [30, 31], which may be facilitated by low TTL. This may explain why TTL was specifically associated with CVD mortality in older adults.

The fact that TTL was not associated with cancer mortality may suggest that this marker gives insights into the oxidative status of blood vessels but not necessarily into the oxidative status of cells (which could degenerate to cancer cells by oxidative DNA damage and other oxidative pathways [32]). On the contrary, d-ROMs serum levels were associated with cancer mortality, implying that they may give information about the ROS concentration in cells. This is not unexpected because it is known that hydroperoxides, which are the ROS class being detected by the d-ROMs assay, can be transported from cells to blood vessels to decrease the oxidative potential of a cell.

### Clinical implications and future research

The presented analyses focused on mortality. Therefore, future studies should shed light on the associations of d-ROMs levels and TTL with major underlying causes of mortality. Only one large-scale application of the d-ROMs assay for the outcome “incident colorectal cancer” is available so far, reporting a strong association with an adjusted incidence rate ratio for comparison of top and bottom d-ROMs tertile of 1.91 (95 % CI, 1.47–2.48) [23]. However, there is a large number of diseases with a potential oxidative stress component that should be addressed in future studies (e.g. CVD, neoplasms, type 2 diabetes mellitus, osteoarthritis, autoimmune diseases, and neurologic diseases) [32]. If relationships could be established, d-ROMs levels and TTL could be used as prognostic markers for such diseases to guide early prevention. In particular, CVD risk prediction in the elderly is a promising target for d-ROMs and TTL measurements because subjects with high d-ROMs levels and low TTL had a 2.5-fold relative risk for CVD mortality compared to subjects with low d-ROMs levels and high TTL in our study. Such a strong effect estimate is remarkable, particularly as it was adjusted for all major established cardiovascular risk factors. Especially in the elderly (≥65 years), in whom traditional cardiovascular risk factors have limited prognostic value [33], oxidative stress markers could be a major cornerstone for cardiovascular risk scores. With control of the oxidative status, frequently mentioned in the media as an important aspect for healthy ageing (mainly to advertise functional foods), there is an increasing demand for testing of the oxidative status in the primary care setting. It is important that this field is evidence-based because, as outlined in the introduction, measuring oxidative stress is complex. This paper has further elaborated the evidence that d-ROMs and TTL assays do measure redox compounds, which can be relevant for the prediction of mortality in middle-aged and older Caucasian individuals.

However, knowing the oxidative status is worth nothing without effective therapies against it. Anti-oxidant supplements have failed to show a reduction of mortality in randomized controlled trials or even increased it [34, 35]. However, this does not discredit the free radical/oxidative stress theory of ageing because it is the proximity of the mitochondrial complex I ROS generating site to the mitochondrial DNA that matters most for ageing [36]. The concentration of anti-oxidants elsewhere is irrelevant. Therefore, since the millennium, treatments targeted against the main enzyme generating ROS, nicotinamide adenine dinucleotide phosphate oxidase (NOX), are being developed with promising results in vivo [37]. However, more research is needed towards isoform-specific NOX-inhibitors but there is optimism that such drugs will be available in the near future [38]. Furthermore, there is still large interest in resveratrol, the active polyphenol present in red wine, which could be cardioprotective, anti-carcinogenic, and anti-inflammatory by potent antioxidant activities [39]. The strong associations of the oxidative stress markers d-ROMs and TTL with mortality in this study show the high potential of anti-oxidative drugs regarding a prolongation of the healthy life-span. Therefore, we can recommend measurement of d-ROMs levels and TTL in clinical trials as surrogate parameters of oxidative stress.

Such clinical trials could also address life-style interventions. It is possible that life-style changes, including smoking cessation, increasing physical activity, weight loss, and a diet favouring nutrients with natural anti-oxidants (e.g. vegetables and fruits) could have favourable effects on oxidative stress marker levels [40]. It is possible that the positive effects of these life-style changes on general health originate in part from their positive effects on redox homeostasis.

Before d-ROMs levels and TTL could be applied as prognostic markers in clinical trials and clinical practice, questions of international standardization of the TTL assay and cut-off values for clinically-relevantly values for both assays should be solved. Although the d-ROMs assay is standardized and suggested cut-offs were suitable for risk stratification in our studies, future studies should elucidate whether sex-specific cut-offs could be better since women have higher d-ROMs levels than men but lower mortality.

## Conclusions

We conclude that this meta-analysis of four population-based cohort studies adds epidemiological evidence to the free radical/oxidative stress theory of ageing. Both d-ROMs levels and TTL were strongly associated with all-cause and CVD mortality and d-ROMs levels were additionally strongly associated with cancer mortality. Noteworthy, individuals with very high d-ROMs levels seem to have a particularly increased risk of short-term mortality, which was approximately 4- to 5-fold increased for CVD and cancer mortality. High d-ROMs levels and low TTL could be markers of different redox pathways associated with pre-mature mortality. Both markers may be useful for cardiovascular risk prediction in the elderly and as surrogate endpoints for clinical trials aiming to evaluate life-style interventions and novel drugs for redox homeostasis.

### Availability of data and materials

No data will be shared because UCL and DKFZ are the owners of the data of the HAPIEE and ESTHER study, respectively, and do not provide open data access for these cohort studies.

## Additional file

Additional file 1:
**Table S1-S8 and Figure S1.** (DOCX 168 kb)

## Abbreviations

Carr U: Carratelli Units
CRP: C-reactive protein
CVD: Cardiovascular disease
d-ROMs: Derivatives of reactive oxygen metabolites
GP: General practitioners
HDL: High-density lipoprotein cholesterol
MI: Myocardial infarction
ROS: Reactive oxygen species
RR: Relative risk
SD: Standard deviation
TTL: Total thiol levels
