# Supplementary material for: Evidence for the free radical/oxidative stress theory of ageing from the CHANCES consortium: a meta-analysis of individual participant data
Source: BMC Med. 2015 Dec 15;13:300. doi: 10.1186/s12916-015-0537-7 (PMC4678534; doi:10.1186/s12916-015-0537-7)
Supplement: Additional file 1: — Table S1-S8 and Figure S1. (DOCX 168 kb) [file 12916_2015_537_MOESM1_ESM.docx]

Supplemental Material


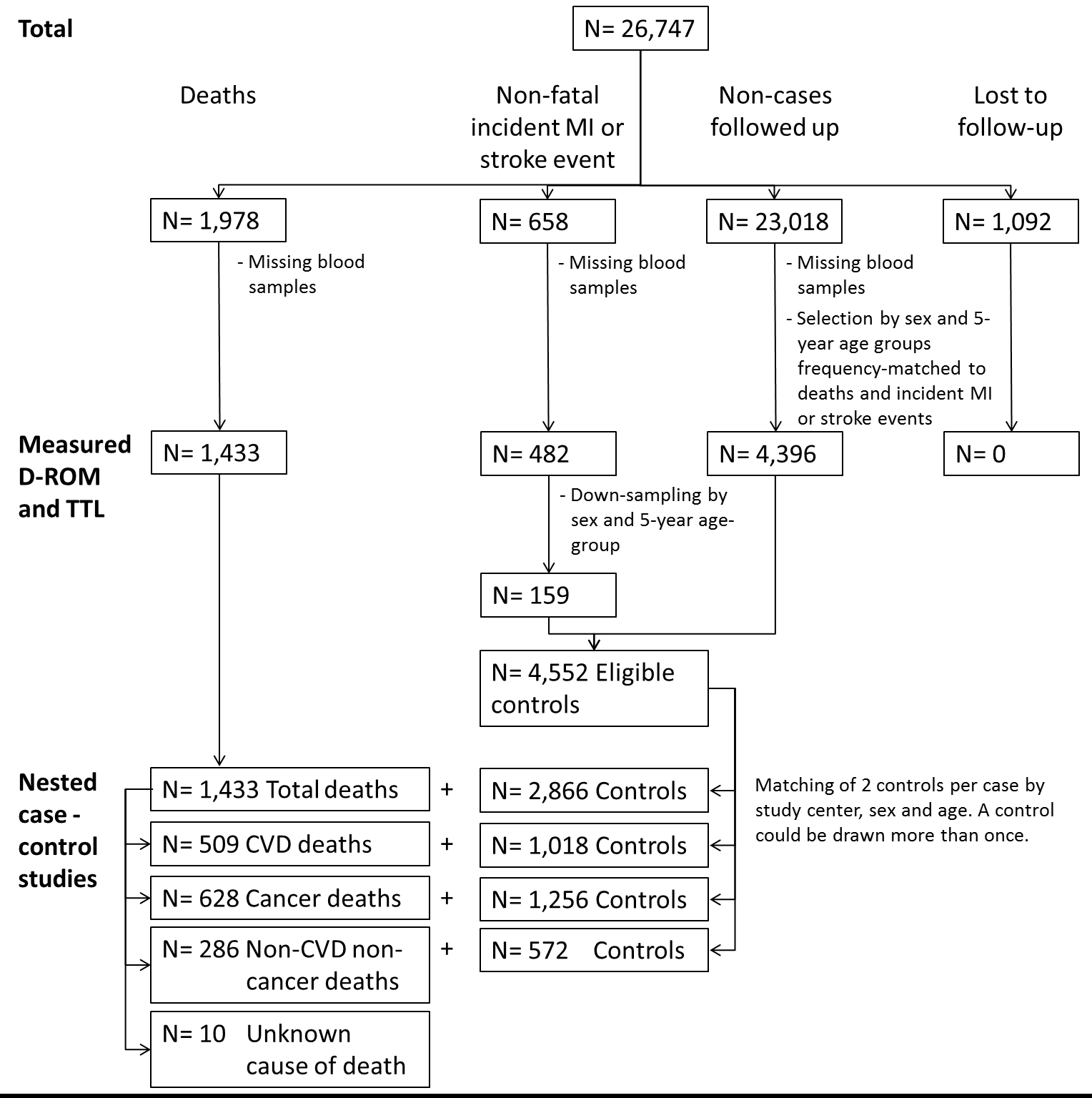


Suppl. Figure 1 – Sampling procedure for the matched case-control desingn in the HAPIEE cohorts

Abbreviations: CVD, cardiovascular disease; D-ROM, derivatives of reactive oxygen metabolites; MI, myocardial infartion; TTL, total thiol levels

**Suppl. Table S1 –** Proportions of missing values in baseline characteristics of study participants by case status (death during follow-up)

| Baseline | Proportion of missing values (%) | | | | |
| --- | --- | --- | --- | --- | --- |
| characteristic | HAPIEE (PL, CZ, LT) | |  | ESTHER | |
|  | Cases  (Deaths) | Eligible  controls |  | Cases  (Deaths) | Controls |
| Total sample size | N=1433 | N=4552 |  | N=269 | N=3758 |
| Age | 0 | 0 |  | 0 | 0 |
| Sex | 0 | 0 |  | 0 | 0 |
| Education | 1 | 1 |  | 3 | 1 |
| BMI | 0 | 0 |  | 4 | 2 |
| Smoking | 1 | 1 |  | 4 | 2 |
| Alcohol consumption | 22 | 21 |  | 7 | 5 |
| Vigorous physical activity | 5 | 3 |  | 22 | 12 |
| Total cholesterol | 0 | 0 |  | 1 | 0 |
| HDL cholesterol | 1 | 1 |  | 1 | 0 |
| CRP | 0 | 0 |  | 0 | 0 |
| eGFR | 0 | 0 |  | 0 | 0 |
| History of hypertension | 1 | 0 |  | 0 | 0 |
| History of diabetes | 1 | 0 |  | 0 | 0 |
| History of MI | 3 | 2 |  | 0 | 0 |
| History of stroke | 3 | 2 |  | 0 | 0 |
| History of cancer | 0 | 0 |  | 0 | 0 |
| D-ROM | 0 | 0 |  | 0 | 0 |
| TTL | 0 | 0 |  | 0 | 0 |

Abbreviations: BMI, body mass index; CRP, C-reactive protein; D-ROM, derivatives of reactive oxygen metabolites; eGFR, estimated glomerular filtration rate; HDL, high-density lipoprotein; MI, myocardial infarction; TTL, total thiol levels.

# Suppl. Table S2 – Cross-sectional determinants of high oxidative stress levels defined by D-ROM levels > 400 Carr U

| **Baseline** | **ESTHER** | **HAPIEE** | **HAPIEE** | **Pooled** | | |
| --- | --- | --- | --- | --- | --- | --- |
| **characteristics** |  | **(Eligible controls)** | **(Cases)** |  | | |
| **Sample size** | **4,027** | **4,552** | **1,433** | **10,012** | **Heterogeneity** | |
| **High oxidative stress** | **938 (23%)** | **1,343 (30%)** | **579 (40%)** | **2,860 (29%)** |  | |
|  | **OR (95%CI)** | **OR (95%CI) ^a^** | **OR (95%CI) ^b^** | **OR (95%CI) ^c^** | **I² (%)** | **P** |
| Age (years) |  |  |  |  |  |  |
| 45 - < 60 | Ref. | Ref. | Ref. | Ref. |  |  |
| 60 - < 65 | 0.81 (0.59; 1.12) | 1.12 (0.91; 1.38) | 1.09 (0.76; 1.56) | 1.03 (0.88; 1.21) | 0 | 0.620 |
| 65 - < 70 | 0.78 (0.57; 1.05) | 0.95 (0.78; 1.15) | **1.47 (1.05; 2.05)** | 1.02 (0.83; 1.25) | 39 | 0.135 |
| 70 - < 85 | 0.98 (0.73; 1.33) | 1.13 (0.79; 1.60) | 1.18 (0.63; 2.19) | 1.06 (0.85; 1.31) | 0 | 0.939 |
| Male sex | **0.19 (0.15; 0.23)** | **0.16 (0.13; 0.20)** | **0.21 (0.12; 0.37)** | **0.18 (0.15; 0.22)** | 35 | 0.160 |
| Education |  |  |  |  |  |  |
| Low | 1.06 (0.72; 1.57) | 0.91 (0.68; 1.21) | 0.95 (0.61; 1.49) | 0.96 (0.78; 1.18) | 0 | 0.865 |
| Medium | 1.17 (0.78; 1.76) | 1.14 (0.95; 1.37) | 0.79 (0.56; 1.11) | 1.07 (0.92; 1.24) | 0 | 0.549 |
| High | Ref. | Ref. | Ref. | Ref. |  |  |
| BMI (kg/m²) |  |  |  |  |  |  |
| < 20 | 1.11 (0.57; 2.18) | 1.14 (0.58; 2.26) | 1.91 (0.58; 6.32) | 1.26 (0.83; 1.92) | 0 | 0.502 |
| 20 - < 25 | Ref. | Ref. | Ref. | Ref. |  |  |
| 25 - < 30 | 0.92 (0.74; 1.15) | **0.77 (0.63; 0.95)** | 0.61 (0.31; 1.21) | **0.77 (0.63; 0.95)** | 45 | 0.093 |
| 30 - < 35 | 0.79 (0.61; 1.02) | **0.66 (0.50; 0.87)** | 0.71 (0.37; 1.34) | **0.71 (0.58; 0.88)** | 31 | 0.188 |
| ≥ 35 | 1.05 (0.75; 1.49) | **0.57 (0.40; 0.79)** | 0.74 (0.28; 1.95) | 0.71 (0.50; 1.03) | 56 | 0.032 |
| Smoking |  |  |  |  |  |  |
| Never | Ref. | Ref. | Ref. | Ref. |  |  |
| Former | 1.18 (0.97; 1.44) | 1.21 (0.88; 1.65) | 1.02 (0.71; 1.48) | **1.15 (1.01; 1.31)** | 0 | 0.460 |
| Current | **1.46 (1.07; 1.98)** | **1.70 (1.38; 2.11)** | **1.61 (1.04; 2.49)** | **1.62 (1.38; 1.90)** | 0 | 0.643 |
| Alcohol consumption ^d^ |  |  |  |  |  |  |
| Abstainer | 1.01 (0.83; 1.22) | 1.05 (0.82; 1.34) | 0.93 (0.65; 1.34) | 1.01 (0.88; 1.16) | 0 | 0.988 |
| Moderate | Ref. | Ref. | Ref. | Ref. |  |  |
| High | 0.79 (0.57; 1.09) | 0.99 (0.77; 1.27) | 0.72 (0.44; 1.15) | 0.88 (0.73; 1.05) | 0 | 0.857 |
| Vigorous physical  activity | 0.96 (0.79; 1.16) | 1.01 (0.85; 1.20) | 1.11 (0.82; 1.49) | 1.00 (0.89; 1.13) | 0 | 0.945 |
| Total cholesterol (mg/dL) |  |  |  |  |  |  |
| < 200 | 0.88 (0.70; 1.10) | **0.76 (0.63; 0.90)** | 0.92 (0.63; 1.36) | **0.83 (0.72; 0.95)** | 14 | 0.322 |
| 200 - < 280 | Ref. | Ref. | Ref. | Ref. |  |  |
| ≥ 280 | **1.35 (1.09; 1.66)** | 1.26 (0.97; 1.63) | 1.28 (0.61; 2.69) | **1.31 (1.09; 1.56)** | 11 | 0.346 |
| HDL cholesterol (mg/dL) |  |  |  |  |  |  |
| < 40 | 1.02 (0.70; 1.51) | **0.71 (0.56; 0.90)** | 1.06 (0.76; 1.49) | 0.85 (0.71; 1.03) | 11 | 0.344 |
| 40 - <80 | Ref. | Ref. | Ref. | Ref. |  |  |
| ≥ 80 | **1.29 (1.02; 1.65)** | 1.28 (0.93; 1.76) | 0.84 (0.48; 1.47) | **1.23 (1.03; 1.48)** | 0 | 0.617 |
| CRP (mg/L) |  |  |  |  |  |  |
| ≤ 3 | Ref. | Ref. | Ref. | Ref. |  |  |
| > 3 - ≤ 10 | **3.21 (2.66; 3.87)** | **3.80 (3.01; 4.81)** | **3.32 (2.10; 5.26)** | **3.51 (2.95; 4.16)** | 44 | 0.096 |
| > 10 | **8.46 (6.22; 11.50)** | **9.01 (6.11; 13.29)** | **9.83 (6.21; 15.54)** | **8.91 (7.27; 10.91)** | 0 | 0.754 |
| Renal impairment ^e^ | 1.17 (0.96; 1.43) | 1.59 (0.88; 2.89) | 0.88 (0.45; 1.72) | 1.24 (0.89; 1.71) | **67** | **0.006** |
| History of hypertension | 1.01 (0.83; 1.23) | **1.23 (1.05; 1.45)** | 1.24 (0.93; 1.66) | **1.15 (1.03; 1.29)** | 0 | 0.731 |
| History of diabetes | 0.96 (0.78; 1.18) | 0.98 (0.73; 1.33) | 0.98 (0.69; 1.38) | 0.98 (0.85; 1.13) | 0 | 0.729 |
| History of MI | 0.99 (0.68; 1.45) | **1.34 (1.01; 1.78)** | 1.30 (0.83; 2.05) | **1.24 (1.02; 1.51)** | 3 | 0.401 |
| History of stroke | 1.13 (0.82; 1.54) | 0.91 (0.60; 1.39) | 1.03 (0.62; 1.69) | 1.04 (0.83; 1.30) | 0 | 0.853 |
| History of cancer | 1.14 (0.90; 1.45) | **1.73 (1.27; 2.38)** | **1.62 (1.07; 2.45)** | **1.51 (1.18; 1.92)** | 35 | 0.164 |

Bold printed: Statistical significant (P<0.05)

Abbreviations: 95%CI: 95% confidence interval; BMI, body mass index; CRP, C-reactive protein; HDL, high-density lipoprotein; MI, myocardial infarction; OR, odds ratio.

^a^ Result of a random effects meta-analysis of ORs from eligible controls of HAPIEE PL, CZ and LT.

^b^ Result of a random effects meta-analysis of ORs from cases of HAPIEE PL, CZ and LT.

^c^ Result of a random effects meta-analysis of ORs from ESTHER, eligible controls of HAPIEE PL, CZ and LT and cases of HAPIEE PL, CZ and LT.

^d^ Definition of moderate alcohol consumption: women >0–19.99 and men >0–39.99 g ethanol per day; and high alcohol consumption: women ≥20 and men ≥40 g

ethanol per day.

^e^ eGFR < 60 mL/min/1.73 m²**Suppl. Table S3** – Cross-sectional determinants of high oxidative stress levels defined by lowest quartile of total thiol levels ^a^

| **Baseline** | **ESTHER** | **HAPIEE** | **HAPIEE** | **Pooled** | | |
| --- | --- | --- | --- | --- | --- | --- |
| **characteristics** |  | **(Eligible controls)** | **(Cases)** |  | | |
| **Sample size** | **4,027** | **4,552** | **1,433** | **10,012** | **Heterogeneity** | |
| **High oxidative stress** | **1,007 (25%)** | **1,141 (25%)** | **429 (30%)** | **2,577 (26%)** |  | |
|  | **OR (95%CI)** | **OR (95%CI) ^b^** | **OR (95%CI) ^c^** | **OR (95%CI) ^d^** | **I² (%)** | **P** |
| Age (years) |  |  |  |  |  |  |
| 45 - < 60 | Ref. | Ref. | Ref. | Ref. |  |  |
| 60 - < 65 | 1.00 (0.72; 1.40) | **1.76 (1.43; 2.16)** | 1.14 (0.73; 1.76) | **1.40 (1.10; 1.78)** | **53** | **0.047** |
| 65 - < 70 | 1.15 (0.84; 1.58) | **2.42 (1.99; 2.93)** | **1.70 (1.23; 2.34)** | **1.88 (1.44; 2.45)** | **66** | **0.007** |
| 70 - < 85 | **1.49 (1.09; 2.02)** | **2.79 (1.44; 5.42)** | 1.91 (0.70; 5.19) | **2.15 (1.37; 3.39)** | **72** | **<0.01** |
| Male sex | **0.72 (0.60; 0.86)** | **0.63 (0.53; 0.75)** | 0.82 (0.53; 1.26) | **0.69 (0.61; 0.77)** | 0 | 0.443 |
| Education |  |  |  |  |  |  |
| Low | 1.01 (0.71; 1.43) | 0.85 (0.35; 2.07) | 0.75 (0.35; 1.63) | 0.83 (0.53; 1.31) | **81** | **<0.01** |
| Medium | 1.02 (0.71; 1.47) | **0.81 (0.69; 0.96)** | 0.80 (0.58; 1.11) | **0.84 (0.73; 0.96)** | 0 | 0.871 |
| High | Ref. | Ref. | Ref. | Ref. |  |  |
| BMI (kg/m²) |  |  |  |  |  |  |
| < 20 | 0.59 (0.26; 1.34) | 1.47 (0.71; 3.05) | 1.29 (0.46; 3.60) | 1.04 (0.64; 1.68) | 0 | 0.467 |
| 20 - < 25 | Ref. | Ref. | Ref. | Ref. |  |  |
| 25 - < 30 | **1.24 (1.00; 1.52)** | **1.28 (1.05; 1.58)** | 1.16 (0.75; 1.81) | **1.25 (1.09; 1.44)** | 0 | 0.994 |
| 30 - < 35 | **1.46 (1.15; 1.86)** | 1.23 (0.88; 1.72) | 1.30 (0.88; 1.93) | **1.34 (1.14; 1.56)** | 2 | 0.408 |
| ≥ 35 | **1.99 (1.45; 2.73)** | **1.73 (1.27; 2.35)** | 1.56 (0.92; 2.64) | **1.80 (1.47; 2.20)** | 0 | 0.783 |
| Smoking |  |  |  |  |  |  |
| Never | Ref. | Ref. | Ref. | Ref. |  |  |
| Former | 1.13 (0.95; 1.35) | 1.13 (0.95; 1.34) | 0.80 (0.57; 1.12) | 1.08 (0.97; 1.22) | 0 | 0.425 |
| Current | **1.45 (1.08; 1.94)** | 1.19 (0.92; 1.54) | 0.69 (0.37; 1.30) | 1.03 (0.78; 1.37) | **67** | **0.006** |
| Alcohol consumption ^e^ |  |  |  |  |  |  |
| Abstainer | 1.10 (0.92; 1.31) | 1.06 (0.81; 1.38) | 1.19 (0.86; 1.64) | 1.10 (0.97; 1.25) | 0 | 0.713 |
| Moderate | Ref. | Ref. | Ref. | Ref. |  |  |
| High | 0.85 (0.62; 1.16) | 0.71 (0.51; 1.00) | 1.07 (0.64; 1.78) | 0.80 (0.64; 1.01) | 24 | 0.245 |
| Vigorous physical  activity | 0.87 (0.74; 1.02) | 1.03 (0.74; 1.45) | 0.95 (0.73; 1.25) | 0.97 (0.83; 1.14) | 46 | 0.083 |
| Total cholesterol (mg/dL) |  |  |  |  |  |  |
| < 200 | 0.97 (0.80; 1.17) | 1.07 (0.91; 1.25) | 1.18 (0.90; 1.54) | 1.05 (0.94; 1.17) | 0 | 0.885 |
| 200 - < 280 | Ref. | Ref. | Ref. | Ref. |  |  |
| ≥ 280 | 0.96 (0.77; 1.19) | 1.06 (0.83; 1.36) | 1.10 (0.70; 1.73) | 1.01 (0.87; 1.18) | 0 | 0.780 |
| HDL cholesterol (mg/dL) |  |  |  |  |  |  |
| < 40 | 1.21 (0.89; 1.64) | 0.92 (0.62; 1.36) | 1.17 (0.86; 1.60) | 1.06 (0.87; 1.28) | 33 | 0.178 |
| 40 - <80 | Ref. | Ref. | Ref. | Ref. |  |  |
| ≥ 80 | 1.04 (0.81; 1.35) | 1.06 (0.77; 1.47) | **1.80 (1.09; 2.97)** | 1.13 (0.94; 1.36) | 0 | 0.496 |
| CRP (mg/L) |  |  |  |  |  |  |
| ≤ 3 | Ref. | Ref. | Ref. | Ref. |  |  |
| > 3 - ≤ 10 | 1.07 (0.90; 1.28) | **1.24 (1.04; 1.47)** | 1.30 (0.99; 1.72) | **1.18 (1.05; 1.32)** | 0 | 0.648 |
| > 10 | **2.63 (1.99; 3.49)** | **2.49 (1.77; 3.52)** | **2.04 (1.38; 3.01)** | **2.44 (2.04; 2.92)** | 0 | 0.553 |
| Renal impairment ^f^ | **2.18 (1.83; 2.60)** | 1.19 (0.92; 1.54) | **1.52 (1.02; 2.28)** | **1.48 (1.10; 2.00)** | **68** | **0.004** |
| History of hypertension | 1.14 (0.95; 1.37) | 1.19 (0.99; 1.42) | 0.87 (0.62; 1.22) | 1.09 (0.95; 1.27) | 37 | 0.146 |
| History of diabetes | 0.99 (0.83; 1.19) | 0.81 (0.65; 1.02) | 1.11 (0.81; 1.53) | 0.95 (0.84; 1.08) | 0 | 0.767 |
| History of MI | 1.27 (0.95; 1.71) | 1.15 (0.89; 1.49) | 1.09 (0.71; 1.69) | 1.17 (0.99; 1.39) | 0 | 0.546 |
| History of stroke | 0.87 (0.66; 1.16) | 0.61 (0.33; 1.12) | 1.14 (0.67; 1.94) | 0.84 (0.62; 1.15) | 38 | 0.140 |
| History of cancer | **1.28 (1.04; 1.59)** | 0.90 (0.66; 1.21) | **1.53 (1.05; 2.23)** | 1.17 (0.94; 1.45) | 28 | 0.216 |

Bold printed: Statistical significant (P<0.05)

Abbreviations: 95%CI: 95% confidence interval; BMI, body mass index; CRP, C-reactive protein; HDL, high-density lipoprotein; MI, myocardial infarction; OR, odds ratio.

^a^ ESTHER < 281.57 μmol/L; HAPIEE PL cases and eligible controls < 464 μmol/L; HAPIEE CZ cases and eligible controls < 371 μmol/L; HAPIEE LT cases and eligible controls < 283 μmol/L.

^b^ Result of a random effects meta-analysis of ORs from eligible controls of HAPIEE PL, CZ and LT.

^c^ Result of a random effects meta-analysis of ORs from cases of HAPIEE PL, CZ and LT.

^d^ Result of a random effects meta-analysis of ORs from ESTHER, eligible controls of HAPIEE PL, CZ and LT and cases of HAPIEE PL, CZ and LT.

^e^ Definition of moderate alcohol consumption: women >0–19.99 and men >0–39.99 g ethanol per day; and high alcohol consumption: women ≥20 and men ≥40 g

ethanol per day.

^f^ eGFR < 60 mL/min/1.73 m²

# Suppl. Table S4 – Cross-sectional association of BMI and HDL with high oxidative stress levels defined by D-ROM levels > 400 Carr U in main model 2 (not adjusted for diseases or CRP)

| **Baseline** | **ESTHER** | **HAPIEE** | **HAPIEE** | **Pooled** ^a^ | | |
| --- | --- | --- | --- | --- | --- | --- |
| **characteristics** |  | **(Eligible controls)** | **(Cases)** ^a^ |  | | |
| **Sample size** | **4,027** | **4,552** | **1,433** | **10,012** | **Heterogeneity** | |
| **High oxidative stress** | **938 (23%)** | **1,343 (30%)** | **579 (40%)** | **2,860 (29%)** |  | |
|  | **OR (95%CI)** | **OR (95%CI) ^b^** | **OR (95%CI) ^c^** | **OR (95%CI) ^d^** | **I² (%)** | **P** |
| BMI (kg/m²) |  |  |  |  |  |  |
| < 20 | 1.14 (0.60; 2.17) | 1.14 (0.59; 2.20) | 1.21 (0.45; 3.26) | 1.15 (0.76; 1.75) | 0 | 0.955 |
| 20 - < 25 | Ref. | Ref. | Ref. | Ref. |  |  |
| 25 - < 30 | 1.07 (0.87; 1.32) | 0.87 (0.72; 1.06) | 0.94 (0.64; 1.36) | 0.96 (0.84; 1.10) | 0 | 0.708 |
| 30 - < 35 | 1.16 (0.92; 1.47) | 0.92 (0.74; 1.15) | 1.23 (0.80; 1.88) | 1.05 (0.90; 1.22) | 0 | 0.436 |
| ≥ 35 | **2.10 (1.55; 2.85)** | 1.18 (0.88; 1.59) | **1.88 (1.11; 3.20)** | **1.53 (1.16; 2.02)** | 41 | 0.128 |
| HDL cholesterol (mg/dL) |  |  |  |  |  |  |
| < 40 | 1.25 (0.87; 1.80) | **0.80 (0.64; 0.99)** | 1.32 (0.93; 1.87) | 1.04 (0.82; 1.32) | 52 | 0.051 |
| 40 - <80 | Ref. | Ref. | Ref. | Ref. |  |  |
| ≥ 80 | 1.10 (0.87; 1.38) | 1.18 (0.81; 1.71) | 0.85 (0.43; 1.69) | 1.09 (0.88; 1.35) | 17 | 0.303 |

Bold printed: Statistical significant (P<0.05)

Abbreviations: 95%CI: 95% confidence interval; BMI, body mass index; HDL, high-density lipoprotein; OR, odds ratio.

^a^ Excluding HAPIEE LT Cases for BMI that had very strong excess risk for low BMI and reduced risk for high BMI.

^b^ Result of a random effects meta-analysis of ORs from eligible controls of HAPIEE PL, CZ and LT.

^c^ Result of a random effects meta-analysis of ORs from cases of HAPIEE PL, CZ and LT.

^d^ Result of a random effects meta-analysis of ORs from ESTHER, eligible controls of HAPIEE PL, CZ and LT and cases of HAPIEE PL, CZ and LT.

**Suppl. Table S5** – Cross-sectional association of education with high oxidative stress levels defined by lowest quartile of TTL ^a^, adjusted for age and sex

| **Baseline** | **ESTHER** | **HAPIEE** | **HAPIEE** | **Pooled** | | |
| --- | --- | --- | --- | --- | --- | --- |
| **characteristics** |  | **(Eligible controls)** | **(Cases)** |  | | |
| **Sample size** | **4,027** | **4,552** | **1,433** | **10,012** | **Heterogeneity** | |
| **High oxidative stress** | **1,007 (25%)** | **1,141 (25%)** | **429 (30%)** | **2,577 (26%)** |  | |
|  | **OR (95%CI)** | **OR (95%CI) ^b^** | **OR (95%CI) ^c^** | **OR (95%CI) ^d^** | **I² (%)** | **P** |
| Education |  |  |  |  |  |  |
| Low | 1.19 (0.85; 1.66) | 0.96 (0.46; 1.99) | 0.85 (0.46; 1.56) | 0.96 (0.66; 1.38) | **74** | **<0.01** |
| Medium | 1.12 (0.79; 1.59) | 0.87 (0.74; 1.03) | 0.82 (0.61; 1.11) | 0.89 (0.78; 1.02) | 0 | 0.817 |
| High | Ref. | Ref. | Ref. | Ref. |  |  |

Abbreviations: 95%CI: 95% confidence interval; OR, odds ratio.

^a^ ESTHER < 281.57 μmol/L; HAPIEE PL cases and eligible controls < 464 μmol/L; HAPIEE CZ cases and eligible controls < 371 μmol/L; HAPIEE LT cases and eligible controls < 283 μmol/L.

^b^ Result of a random effects meta-analysis of ORs from eligible controls of HAPIEE PL, CZ and LT.

^c^ Result of a random effects meta-analysis of ORs from cases of HAPIEE PL, CZ and LT.

^d^ Result of a random effects meta-analysis of ORs from ESTHER, eligible controls of HAPIEE PL, CZ and LT and cases of HAPIEE PL, CZ and LT.

# Suppl. Table S6 – Cross-sectional association of sex (male vs. female) with high oxidative stress levels in models with increasing adjustment

| Model | Definition of high oxidative stress by D-ROM ^a^ | Definition of high oxidative stress by TTL ^b^ |
| --- | --- | --- |
|  | OR (95%CI) ^c^ | OR (95%CI) ^c^ |
| Age-adjusted | 0.22 (0.18; 0.27) | 0.67 (0.61; 0.74) |
| 1 ^d^ | 0.20 (0.16; 0.24) | 0.68 (0.61; 0.76) |
| 2 ^e^ | 0.20 (0.17; 0.25) | 0.67 (0.60; 0.75) |
| 3 ^f^ | 0.19 (0.16; 0.23) | 0.68 (0.61; 0.77) |
| 4 ^g^ | 0.18 (0.15; 0.22) | 0.69 (0.61; 0.77) |

^a^ D-ROM levels > 400 Carr U

^b^ ESTHER < 281.57 μmol/L; HAPIEE PL cases and eligible controls < 464 μmol/L; HAPIEE CZ cases and eligible controls < 371 μmol/L; HAPIEE LT cases and eligible controls < 283 μmol/L.

^c^ Adjusted for age, sex (matched for HAPIEE), education, BMI, smoking, alcohol consumption and vigorous physical activity.

^d^ Result of a random effects meta-analysis of ORs from ESTHER, eligible controls of HAPIEE PL, CZ and LT and cases of HAPIEE PL, CZ and LT.

^e^ Adjusted for variable of model 1 plus total cholesterol and HDL cholesterol.

^f^ Adjusted for variable of model 2 plus renal impairment, history of diabetes, history of myocardial infarction, history of stroke and history of cancer.

^g^ Adjusted for variable of model 3 plus CRP.

**Suppl. Table S7** – Cross-sectional determinants of high oxidative stress levels (defined by D-ROM levels > 400 Carr U, stratified by sex

| **Baseline** | **ESTHER** | |  | **HAPIEE Eligible Controls** | |
| --- | --- | --- | --- | --- | --- |
| **characteristics** | **Women** | **Men** |  | **Women** | **Men** |
| **Total sample size** | **2,233** | **1,622** |  | **1,537** | **3,015** |
| **High oxidative stress** | **766 (34%)** | **172 (10%)** |  | **794 (52%)** | **549 (18%)** |
|  | **OR (95%CI)** | **OR (95%CI)** |  | **OR (95%CI) ^a^** | **OR (95%CI) ^a^** |
| Age (years) |  |  |  |  |  |
| 45 - < 60 | Ref. | Ref. |  | Ref. | Ref. |
| 60 - < 65 | 0.76 (0.53; 1.09) | 1.11 (0.49; 2.52) |  | 1.11 (0.81; 1.51) | 1.12 (0.85; 1.48) |
| 65 - < 70 | **0.70 (0.51; 0.99)** | 1.15 (0.53; 2.51) |  | 0.93 (0.67; 1.24) | 0.92 (0.70; 1.21) |
| 70 - < 85 | 0.86 (0.62; 1.20) | 1.65 (0.78; 3.49) |  | 0.82 (0.52; 1.29) | 1.37 (0.90; 2.08) |
| Education |  |  |  |  |  |
| Low | 1.14 (0.71; 1.82) | 0.88 (0.45; 1.75) |  | 0.71 (0.49; 1.05) | 1.22 (0.80; 1.86) |
| Medium | 1.25 (0.76; 2.05) | 1.04 (0.50; 2.14) |  | 1.00 (0.76; 1.31) | 1.23 (0.96; 1.57) |
| High | Ref. | Ref. |  | Ref. | Ref. |
| BMI (kg/m²) |  |  |  |  |  |
| < 20 | 1.26 (0.62; 2.53) | Not estimable |  | 0.47 (0.14; 1.59) | 1.66 (0.75; 3.66) |
| 20 - < 25 | Ref. | Ref. |  | Ref. | Ref. |
| 25 - < 30 | 0.96 (0.75; 1.23) | 0.85 (0.53; 1.34) |  | 0.83 (0.61; 1.13) | 0.76 (0.58; 1.01) |
| 30 - < 35 | 0.91 (0.68; 1.21) | 0.57 (0.33; 1.01) |  | 0.89 (0.63; 1.26) | **0.55 (0.39; 0.76)** |
| ≥ 35 | 1.01 (0.68; 1.49) | 1.42 (0.71; 2.82) |  | 0.80 (0.51; 1.26) | **0.40 (0.24; 0.68)** |
| Smoking |  |  |  |  |  |
| Never | Ref. | Ref. |  | Ref. | Ref. |
| Former | 1.25 (1.00; 1.57) | 1.09 (0.75; 1.58) |  | 1.10 (0.80; 1.51) | 1.22 (0.95; 1.58) |
| Current | 1.32 (0.92; 1.89) | **1.86 (1.02; 3.40)** |  | 1.22 (0.88; 1.69) | **2.02 (1.53; 2.66)** |
| Alcohol consumption ^b^ |  |  |  |  |  |
| Abstainer | 0.97 (0.78; 1.20) | 1.24 (0.77; 1.98) |  | 0.94 (0.69; 1.28) | 1.20 (0.78; 1.84) |
| Moderate | Ref. | Ref. |  | Ref. | Ref. |
| High | 0.78 (0.54; 1.13) | 0.78 (0.36; 1.70) |  | 1.04 (0.54; 2.02) | 0.99 (0.74; 1.33) |
| Vigorous physical  activity | 0.92 (0.74; 1.15) | 1.05 (0.69; 1.61) |  | 1.00 (0.77; 1.28) | 1.03 (0.82; 1.30) |
| Total cholesterol (mg/dL) |  |  |  |  |  |
| < 200 | 0.92 (0.70; 1.23) | 0.81 (0.55; 1.19) |  | 0.77 (0.59; 1.02) | **0.74 (0.59; 0.93)** |
| 200 - < 280 | Ref. | Ref. |  | Ref. | Ref. |
| ≥ 280 | **1.30 (1.03; 1.63)** | 1.73 (0.98; 3.04) |  | **1.48 (1.05; 2.11)** | 1.07 (0.73; 1.57) |
| HDL cholesterol (mg/dL) |  |  |  |  |  |
| < 40 | 1.12 (0.61; 2.06) | 0.92 (0.54; 1.56) |  | 0.60 (0.39; 0.91) | 0.78 (0.60; 1.03) |
| 40 - <80 | Ref. | Ref. |  | Ref. | Ref. |
| ≥ 80 | **1.31 (1.02; 1.69)** | 0.89 (0.32; 2.45) |  | 1.19 (0.81; 1.76) | 1.32 (0.78; 2.24) |
| CRP (mg/L) |  |  |  |  |  |
| ≤ 3 | Ref. | Ref. |  | Ref. | Ref. |
| > 3 - ≤ 10 | **2.94 (2.37; 3.65)** | **4.19 (2.86; 6.16)** |  | **3.19 (2.44; 4.18)** | **4.16 (3.31; 5.20)** |
| > 10 | **6.96 (4.67; 10.37)** | **12.83 (7.81; 21.07)** |  | **5.92 (3.30; 10.64)** | **11.25 (7.66; 16.53)** |
| Renal impairment ^c^ | 1.16 (0.92; 1.45) | 1.16 (0.76; 1.79) |  | 1.35 (0.89; 2.03) | **1.68 (1.16; 2.43)** |
| History of hypertension | 1.06 (0.85; 1.33) | 0.87 (0.58; 1.31) |  | 1.21 (0.95; 1.54) | 1.23 (0.99; 1.53) |
| History of diabetes | 0.91 (0.72; 1.17) | 1.10 (0.75; 1.62) |  | 0.77 (0.53; 1.11) | 1.21 (0.89; 1.64) |
| History of MI | 1.08 (0.61; 1.89) | 0.89 (0.52; 1.53) |  | 1.04 (0.60; 1.80) | **1.50 (1.08; 2.07)** |
| History of stroke | 1.20 (0.81; 1.76) | 0.95 (0.55; 1.65) |  | 1.00 (0.52; 1.92) | 0.82 (0.47; 1.41) |
| History of cancer | 1.07 (0.80; 1.42) | 1.39 (0.89; 2.19) |  | **1.55 (1.01; 2.37)** | **1.83 (1.19; 2.80)** |

Bold printed: Statistical significant (P<0.05)

Abbreviations: 95%CI: 95% confidence interval; BMI, body mass index; CRP, C-reactive protein; HDL, high-density lipoprotein; MI, myocardial infarction; OR, odds ratio.

^a^ Result of a random effects meta-analysis of ORs from eligible controls of HAPIEE PL, CZ and LT.

^b^ Definition of moderate alcohol consumption: women >0–19.99 and men >0–39.99 g ethanol per day; and high alcohol consumption: women ≥20 and men ≥40 g

ethanol per day.

^c^ eGFR < 60 mL/min/1.73 m²

**Suppl. Table S8** – Cross-sectional determinants of high oxidative stress levels lowest quartile of total thiol levels ^a^, stratified by sex

| **Baseline** | **ESTHER** | |  | **HAPIEE Eligible Controls** | |
| --- | --- | --- | --- | --- | --- |
| **characteristics** | **Women** | **Men** |  | **Women** | **Men** |
| **Total sample size** | **2,233** | **1,622** |  | **1,537** | **3,015** |
| **High oxidative stress** | **612 (27%)** | **395 (22%)** |  | **473 (31%)** | **2,347 (22%)** |
|  | **OR (95%CI)** | **OR (95%CI)** |  | **OR (95%CI) ^b^** | **OR (95%CI) ^b^** |
| Age (years) |  |  |  |  |  |
| 45 - < 60 | Ref. | Ref. |  | Ref. | Ref. |
| 60 - < 65 | 1.13 (0.74; 1.72) | 0.80 (0.45; 1.40) |  | **1.68 (1.20; 2.36)** | **1.73 (1.34; 2.23)** |
| 65 - < 70 | 1.09 (0.73; 1.62) | 1.13 (0.67; 1.91) |  | **2.66 (1.93; 3.65)** | **2.17 (1.71; 2.76)** |
| 70 - < 85 | **1.71 (1.16; 2.52)** | 1.10 (0.66; 1.83) |  | **3.54 (2.23; 5.61)** | **2.24 (1.57; 3.20)** |
| Education |  |  |  |  |  |
| Low | 0.96 (0.58; 1.60) | 1.06 (0.66; 1.71) |  | 0.87 (0.60; 1.28) | 0.87 (0.61; 1.24) |
| Medium | 1.01 (0.59; 1.72) | 1.04 (0.63; 1.72) |  | 0.99 (0.74; 1.33) | **0.78 (0.64; 0.95)** |
| High | Ref. | Ref. |  | Ref. | Ref. |
| BMI (kg/m²) |  |  |  |  |  |
| < 20 | 0.51 (0.20; 1.31) | 1.17 (0.22; 6.10) |  | 0.45 (0.09; 2.11) | 1.58 (0.74; 3.41) |
| 20 - < 25 | Ref. | Ref. |  | Ref. | Ref. |
| 25 - < 30 | **1.43 (1.10; 1.88)** | 0.96 (0.68; 1.34) |  | 1.17 (0.84; 1.63) | 1.26 (0.97; 1.62) |
| 30 - < 35 | **1.57 (1.15; 2.15)** | 1.21 (0.83; 1.77) |  | 1.21 (0.84; 1.75) | 1.17 (0.88; 1.57) |
| ≥ 35 | **2.54 (1.70; 3.80)** | 1.25 (0.73; 2.13) |  | 1.41 (0.90; 2.22) | **1.94 (1.28; 2.93)** |
| Smoking |  |  |  |  |  |
| Never | Ref. | Ref. |  | Ref. | Ref. |
| Former | 1.08 (0.84; 1.31) | 1.21 (0.94; 1.56) |  | 1.07 (0.78; 1.48) | 1.16 (0.94; 1.43) |
| Current | **1.60 (1.09; 2.37)** | 1.26 (0.80; 1.99) |  | 1.28 (0.91; 1.81) | 1.21 (0.94; 1.43) |
| Alcohol consumption ^c^ |  |  |  |  |  |
| Abstainer | 1.05 (0.84; 1.31) | 1.14 (0.84; 1.54) |  | 1.41 (0.90; 2.20) | 0.84 (0.65; 1.09) |
| Moderate | Ref. | Ref. |  | Ref. | Ref. |
| High | 0.87 (0.59; 1.29) | 0.88 (0.53; 1.47) |  | 0.99 (0.59; 1.64) | **0.63 (0.48; 0.83)** |
| Vigorous physical  activity | **0.80 (0.65; 0.99)** | 1.01 (0.78; 1.31) |  | 1.08 (0.84; 1.39) | 1.01 (0.82; 1.23) |
| Total cholesterol (mg/dL) |  |  |  |  |  |
| < 200 | 1.04 (0.78; 1.38) | 0.93 (0.71; 1.20) |  | 1.02 (0.77; 1.35) | 1.09 (0.90; 1.32) |
| 200 - < 280 | Ref. | Ref. |  | Ref. | Ref. |
| ≥ 280 | 1.00 (0.77; 1.28) | 0.89 (0.56; 1.41) |  | 1.05 (0.74; 1.48) | 1.12 (0.80; 1.57) |
| HDL cholesterol (mg/dL) |  |  |  |  |  |
| < 40 | 0.89 (0.48; 1.64) | 1.37 (0.96; 1.97) |  | 0.68 (0.43; 1.06) | 1.08 (0.86; 1.36) |
| 40 - <80 | Ref. | Ref. |  | Ref. | Ref. |
| ≥ 80 | 1.19 (0.90; 1.56) | 0.54 (0.24; 1.23) |  | 1.04 (0.69; 1.56) | 0.94 (0.57; 1.56) |
| CRP (mg/L) |  |  |  |  |  |
| ≤ 3 | Ref. | Ref. |  | Ref. | Ref. |
| > 3 - ≤ 10 | 1.13 (0.89; 1.42) | 0.99 (0.74; 1.31) |  | 1.35 (1.04; 1.75) | 1.16 (0.93; 1.44) |
| > 10 | **2.43 (1.65; 3.56)** | **2.75 (1.81; 4.19)** |  | **2.40 (1.47; 3.92)** | **2.30 (1.60; 3.30)** |
| Renal impairment ^d^ | **1.93 (1.54; 2.42)** | **2.61 (1.96; 3.48)** |  | 1.22 (0.83; 1.79) | 1.23 (0.88; 1.72) |
| History of hypertension | **1.27 (1.00; 1.62)** | 0.99 (0.74; 1.33) |  | 1.16 (0.91; 1.48) | 1.19 (0.98; 1.44) |
| History of diabetes | 1.03 (0.81; 1.32) | 0.95 (0.73; 1.24) |  | 0.71 (0.49; 1.04) | 0.88 (0.67; 1.15) |
| History of MI | **1.78 (1.03; 3.08)** | 1.18 (0.83; 1.70) |  | 1.00 (0.65; 1.72) | 1.12 (0.84; 1.49) |
| History of stroke | 0.86 (0.57; 1.29) | 0.92 (0.62; 1.36) |  | 0.93 (0.50; 1.76) | **0.53 (0.31; 0.89)** |
| History of cancer | **1.41 (1.06; 1.88)** | 1.18 (0.86; 1.63) |  | 1.01 (0.65; 1.55) | 0.90 (0.60; 1.35) |

Bold printed: Statistical significant (P<0.05)

Abbreviations: 95%CI: 95% confidence interval; BMI, body mass index; CRP, C-reactive protein; HDL, high-density lipoprotein; MI, myocardial infarction; OR, odds ratio.

^a^ ESTHER < 281.57 μmol/L; HAPIEE PL cases and eligible controls < 464 μmol/L; HAPIEE CZ cases and eligible controls < 371 μmol/L; HAPIEE LT cases and eligible controls < 283 μmol/L.

^b^ Result of a random effects meta-analysis of ORs from eligible controls of HAPIEE PL, CZ and LT.

^c^ Definition of moderate alcohol consumption: women >0–19.99 and men >0–39.99 g ethanol per day; and high alcohol consumption: women ≥20 and men ≥40 g

ethanol per day.

^d^ eGFR < 60 mL/min/1.73 m²
